# A Multiscale Closed-Loop Neurotoxicity Model of Alzheimer’s Disease Progression Explains Functional Connectivity Alterations

**DOI:** 10.1523/ENEURO.0345-23.2023

**Published:** 2024-04-12

**Authors:** Jesús Cabrera-Álvarez, Leon Stefanovski, Leon Martin, Gianluca Susi, Fernando Maestú, Petra Ritter

**Affiliations:** ^1^Department of Experimental Psychology, Complutense University of Madrid, Pozuelo de Alarcón 28223, Spain; ^2^Centre for Cognitive and Computational Neuroscience, Complutense University of Madrid, Madrid 28040, Spain; ^3^Berlin Institute of Health at Charité - Universitätsmedizin Berlin, Berlin 10117, Germany; ^4^Department of Neurology with Experimental Neurology, Brain Simulation Section, Charité - Universitätsmedizin Berlin, Berlin 10117, Germany; ^5^Department of Structure of Matter, Thermal Physics and Electronics, Complutense University of Madrid, Madrid 28040, Spain; ^6^Bernstein Center for Computational Neuroscience Berlin, Berlin 10115, Germany

**Keywords:** Alzheimer’s disease, functional connectivity, Jansen–Rit, multiscale modeling, proteinopathy, simulation

## Abstract

The accumulation of amyloid-*β* (*Aβ*) and hyperphosphorylated-tau (hp-tau) are two classical histopathological biomarkers in Alzheimer’s disease (AD). However, their detailed interactions with the electrophysiological changes at the meso- and macroscale are not yet fully understood. We developed a mechanistic multiscale model of AD progression, linking proteinopathy to its effects on neural activity and vice-versa. We integrated a heterodimer model of prion-like protein propagation and a brain network model of Jansen–Rit neural masses derived from human neuroimaging data whose parameters varied due to neurotoxicity. Results showed that changes in inhibition guided the electrophysiological alterations found in AD, and these changes were mainly attributed to *Aβ* effects. Additionally, we found a causal disconnection between cellular hyperactivity and interregional hypersynchrony contrary to previous beliefs. Finally, we demonstrated that early *Aβ* and hp-tau depositions’ location determine the spatiotemporal profile of the proteinopathy. The presented model combines the molecular effects of both *Aβ* and hp-tau together with a mechanistic protein propagation model and network effects within a closed-loop model. This holds the potential to enlighten the interplay between AD mechanisms on various scales, aiming to develop and test novel hypotheses on the contribution of different AD-related variables to the disease evolution.

## Significance Statement

This research presents a closed-loop model of Alzheimer’s disease (AD) mechanisms, bridging the gap between protein distribution and neural activity. Contrary to prior beliefs, the study reveals that interregional hypersynchrony and cellular hyperactivity are not directly linked. Notably, the model identifies neural inhibition as a potential causal factor in neurophysiological AD alterations and posits early depositions of *Aβ* as a determinant of the spatiotemporal profile of proteinopathy. The significance of this mechanistic disease framework lies in its potential to produce insights into AD evolution and to guide novel treatment strategies. It underscores the importance of further experiments and modeling efforts to refine our understanding of AD, offering hope for more effective treatments and personalized care in the fight against dementia.

## Introduction

Alzheimer’s disease (AD) is a progressive neurodegenerative disorder widely characterized at several levels of analysis from molecules to cells to the whole brain. The interactions between these levels of analysis remain poorly understood, contributing to the limited treatment options (Breijyeh and Karaman, 2020; van Dyck et al., 2023). A mechanistic understanding of AD is essential for developing novel therapeutic interventions.

The main pathophysiological changes in AD involve the misfolding and accumulation of amyloid-*β* (*Aβ*) and a hyperphosphorylated version of the tau protein (hp-tau) in the brain (Masters et al., 2015). These two proteins have different toxic effects, while *Aβ* generates hyperactivity through the disruption of GABAergic inhibitory synapses (Palop and Mucke, 2010) and the reduction of glutamate reuptake (Zott et al., 2019), hp-tau disrupts the synaptic connectivity of neurons by reducing the number of dendritic spines in pyramidal cells (Merino-Serrais et al., 2013). *Aβ* tends to aggregate into plaques, while hp-tau aggregates into neurofibrillary tangles (NFTs). Following the prion hypothesis of AD, misfolded *Aβ* and hp-tau propagate through the brain acting as seeds (i.e., prions) that trigger the misfolding and aggregation of their normal counterparts (Frost and Diamond, 2009; Walker, 2018; Gomez-Gutierrez and Morales, 2020). It has been shown that the hyperactivity produced by *Aβ* orients the prionic propagation of hp-tau in the brain (Rodriguez et al., 2020) following the Braak stages from the entorhinal cortex to the neocortex, producing a network disruption that has been linked to cognitive decline (Braak and Braak, 1991; Braak et al., 2011; Therriault et al., 2022). The higher the activity levels in a region, the faster the propagation of hp-tau to those regions. Additionally, hyperactivation has also been linked to an enhanced extracellular secretion and deposition of *Aβ* generating a cyclical phenomenon that reinforces bidirectionally neural hyperactivity and *Aβ* concentration (Kamenetz et al., 2003; Cirrito et al., 2005; Stargardt et al., 2015; Yamamoto et al., 2015; Busche and Hyman, 2020).

At the whole brain level, electrophysiological recordings have shown a slowing of *α* frequency peak along the disease evolution (Babiloni et al., 2009; Garcés et al., 2013). Additionally, several studies have reported an early increase in *α* power and hypersynchrony between parietal regions (Nakamura et al., 2017, 2018), followed by a disruption in those network’s functional connectivity (FC) and a rise in anterior hypersynchrony (López-Sanz et al., 2016; Pusil et al., 2019). These observations suggest a temporal dissociation between anterior and posterior in the electrophysiological changes that occur in AD (Maestú et al., 2021). Finally, the whole network gets disrupted due to the effect of hp-tau accumulation and further generation of NFTs (Palop et al., 2006; Ranasinghe et al., 2014; Ahnaou et al., 2017; Pereira et al., 2019). Whether the hypersynchronization is linked to the previously mentioned cellular hyperactivity remains unknown (Maestú et al., 2021). Here, we hypothesize that hyperactivity causes an increase in interregional synchrony (i.e., FC) due to a higher rate of neural communication that enhances the coordination of distant neuronal groups.

In recent years, significant computational modeling efforts have employed brain network models (BNMs) to investigate neural activity and its relationship to several aspects of the disease (Stefanovski et al., 2019, 2021; Ranasinghe et al., 2022; van Nifterick et al., 2022; Alexandersen et al., 2023). These models reproduce neural activity using sets of differential equations known as neural mass models (NMMs) that are interconnected through empirically derived structural connectivity (SC) networks based on tractography. These models are expected to serve as a tool for getting deeper insights into the mechanisms of AD and to be able to predict its appearance and evolution.

In this study, we extended a previously published multiscale model of AD evolution based on neuroimaging data that includes molecular, cellular, and interregional features of the brain (Alexandersen et al., 2023). Specifically, it integrates a heterodimer model for the generation and propagation of AD-related proteins, neuronal population activity, and interregional synaptic coupling. We aimed to explore the mechanisms that give rise to the observed neurophysiological changes in AD and to evaluate the relative impact of the different proteins involved. To pursue these goals, first, we explored an isolated neural population and the effects produced by changing the parameter candidates affected by AD (i.e., excitation and inhibition-related parameters). Second, we simulated the neurotoxicity model and adjusted its parameters to reproduce the empirically observed phenomena in AD (i.e., frequency slowing, relative *α* decrease, increase/decrease in excitation, and increase/decrease in FC). Finally, we use the resulting network model to analyze the impact of different biological mechanisms giving rise to AD progression. The mechanistic explanations derived from the model are key to understanding the effects of delivered treatments, fostering the development of new ones, and enhancing early detection methods.

## Methods

### Data acquisition

Magnetic resonance imaging (MRI) scans were acquired for 20 healthy participants (17 females) with mean age 60.5 (sd 4.17) at the Centre for Cognitive and Computational Neuroscience, Complutense University of Madrid. Diffusion-weighted images (dw-MRI) were acquired with a single-shot echo-planar imaging sequence with the parameters: echo time/repetition time = 96.1/12,000 ms, NEX 3 for increasing the signal-to-noise ratio, slice thickness = 2.4 mm, 128 × 128 matrix, and field of view = 30.7 cm yielding an isotropic voxel of 2.4 mm; 1 image with no diffusion sensitization (i.e., T2-weighted b0 images) and 25 dw-MRI (*b* = 900 s/mm^2^).

All participants provided informed consent.

### SC

In a preprocessing stage, FSL eddy was used to correct for eddy current distortion. The correction was conducted through the integrated interface in DSI Studio (http://dsi-studio.labsolver.org). Then, the eddy-corrected dw-MRI data were rotated to align with the antero-posterior commisure line. The restricted diffusion was quantified using restricted diffusion imaging (Yeh et al., 2016). The diffusion data were reconstructed using generalized q-sampling imaging (Yeh et al., 2010) with a diffusion sampling length ratio of 1.25. The tensor metrics were calculated using dw-MRI with a *b*-value lower than 1750 s/mm^2^. A deterministic fiber tracking algorithm (Yeh et al., 2013) was used with augmented tracking strategies (Yeh, 2020) to improve reproducibility. The whole brain was used as seeding region. The anisotropy threshold was randomly selected. The angular threshold was randomly selected from 15° to 90°. The step size was randomly selected from 0.5 voxels to 1.5 voxels. Tracks with lengths shorter than 15 or longer than 300 mm were discarded. A total of 5 million seeds were placed.

**Figure 1.**
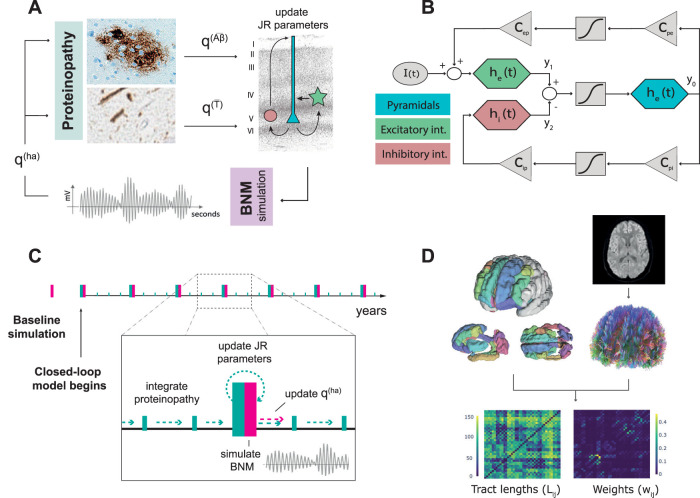
Modeling framework overview. ***A***, The closed-loop neurotoxicity model integrates a model of proteinopathy dynamics and a BNM to simulate neural activity, interacting in a closed-loop: proteinopathy affects neural activity and vice-versa. ***B***, Scheme of the Jansen–Rit (JR) NMM with three interacting subpopulations where triangles represent synaptic contacts between subpopulations, boxes represent the sigmoidal transformation of voltage into firing rate, and colored hexagons represent the transformation of firing rate into voltage. ***C***, Implementation timeline for the closed-loop model. First, a baseline simulation is gathered to get a baseline firing rate. Then, the closed-loop model simulation begins by integrating the proteinopathy with *dt* = 0.25 (years) and simulating the BNM to update hyperactivity damage ($q_{i}^{\left(ha\right)}$) with *dt* = 1 (year). ***D***, Pipeline to extract SC matrices. Tractography is performed on dw-MRI data, and SC matrices are extracted using a customized version of the DK atlas. The histological image for the *Aβ* plaque in panel ***A*** was distributed by Michael Bonert, under a CC BY-SA 3.0 license. The NFT image was taken from Moloney et al. (2021).

A combination of Desikan–Killiany (Desikan et al., 2006) and ASEG (Fischl et al., 2002) atlases were used as a volume parcellation atlas, and two SC matrices were calculated (Fig. 1*D*): weights using the count of the connecting tracks, and tract length using the average length of the streamlines connecting two regions. The final SC consisted of a subset of these networks including 40 regions (Table 3) representing the cingulum bundle (Bubb et al., 2018), one of the most prominent white matter structures that interconnect frontal, parietal, temporal, and subcortical regions, and whose impairment has been related to AD and cognitive decline (Yu et al., 2017; Bubb et al., 2018; Chen et al., 2023).

### BNM

SC matrices served as the skeleton for the BNMs implemented in the virtual brain (Sanz-Leon et al., 2015) where regional signals were simulated using JR NMMs (Jansen and Rit, 1995). The JR is a biologically inspired model of a cortical column capable of reproducing *α* oscillations through a system of second-order coupled differential equations (Table 1 for a description of parameters; Fig. 1*B*):

(1) $${\dot{y}}_{0_{i}}=y_{3_{i}},$$

(2) $${\dot{y}}_{1_{i}}=y_{4_{i}},$$

(3) $${\dot{y}}_{2_{i}}=y_{5_{i}},$$

(4) $${\dot{y}}_{3_{i}}=\frac{H_{e}}{\tau_{e}}\;S[\;y_{1_{i}}-y_{2_{i}}\;]-\frac{2}{\tau_{e}}y_{3_{i}}-\frac{1}{\tau_{e}^{2}}y_{0_{i}},$$

(5) $${\dot{y}}_{4_{i}}=\frac{H_{e}}{\tau_{e}}\;\{\;I_{i}(t)+C_{ep}\;S[C_{\;pe}\;y_{0_{i}}]\;\}-\frac{2}{\tau_{e}}y_{4_{i}}-\frac{1}{\tau_{e}^{2}}y_{1_{i}},$$

(6) $${\dot{y}}_{5_{i}}=\frac{H_{i}}{\tau_{i}}\;C_{ip}\;S[\;C_{\;pi}\;y_{0_{i}}\;]-\frac{2}{\tau_{i}}y_{5_{i}}-\frac{1}{\tau_{i}^{2}}y_{2_{i}},$$

where

(7) $$S[v]=\frac{2\;e_{0}}{1+e^{r(v_{0}-v)}},$$

(8) $$I_{i}(t)=\eta_{i}(t)+g\sum_{\;j=1}^{n}w_{\;ji}\;S[y_{1_{j}}(t-d_{\;ji})-y_{2_{j}}(t-d_{\;ji})]$$

for *i* = 1, …, *N* with *N* as the number of simulated regions. The interregional communication introduces heterogeneity in terms of connection strength *w*_*ji*_, and conduction delays *d*_*ji*_ between nodes *i* and *j* where *d*_*ji*_ = *L*_*ji*_/*s*, with *L*_*ji*_ being the length of the tract from node *i* to node *j*, and *s* representing the conduction speed.

**Table 1.** JR-BNM parameters used in simulations

Parameter	Value	Unit	Description
*H*_*e*_	3.25	mV	Average excitatory synaptic gain
*H*_*i*_	22	mV	Average inhibitory synaptic gain
$\tau_{e}$	10	ms	Time constant of excitatory PSP
$\tau_{i}$	20	ms	Time constant of inhibitory PSP
*C*_*pe*_	135		Average synaptic contacts: pyramidals to excitatory interneurons
*C*_*ep*_	108		Average synaptic contacts: excitatory interneurons to pyramidals
*C*_*pi*_	33.75		Average synaptic contacts: pyramidals to inhibitory interneurons
*C*_*ip*_	33.75		Average synaptic contacts: inhibitory interneurons to pyramidals
*e*_0_	0.0025	ms^−1^	Half the maximum firing rate
*r*	0.56	mV^−1^	Slope of the presynaptic function at *v*_0_
*v*_0_	6	mV	Potential when half the maximum firing rate is achieved
*p*	0.1085	ms^−1^	Mean of random Gaussian intrinsic noisy input
*σ*	0.022	ms^−1^	Standard deviation of random Gaussian intrinsic noisy input
*g*	25		Coupling factor for interregional communication
*s*	20	m/s	Conduction speed for interregional communication

This model represents the electrophysiological activity (in voltage) of three subpopulations of neurons: pyramidal neurons (*y*_0_), excitatory interneurons (*y*_1_), and inhibitory interneurons (*y*_2_) and their derivatives (*y*_3_, *y*_4_, and *y*_5_, respectively). These subpopulations are interconnected through an average number of synaptic contacts (*C*_*ep*_, *C*_*ip*_, *C*_*pe*_, and *C*_*pi*_), and integrate external inputs from other cortical columns. The communication is implemented in terms of firing rate, using a sigmoidal function for the conversion of the voltage inputs (Eq. 7). Parameters regarding postsynaptic potential amplitudes (*H*_*e*_, *H*_*i*_) and time constants ($\tau_{e},\tau_{i}$) shape the oscillatory behavior of subpopulations’ voltages.

The input (*I*_*i*_(*t*)) represents two main drivers of activity in the NMMs: interregional communication and an intrinsic noisy input. The latter is defined as a local and independent Gaussian noise $\eta_{i}(t)∼N(p,\sigma )$.

A global coupling factor *g* is implemented to scale linearly tracts’ weights. Both *g* and *s* are scaling factors that apply to all nodes and were adjusted to reproduce the qualitative features of the AD evolution.

### Closed-loop neurotoxicity model

We have adapted an already published model for AD protein propagation, first shown by Thompson et al. (2020), that reproduces the evolution of tau and *Aβ* concentrations in the AD brain, and a further extension by Alexandersen et al. (2023) that linked this evolution to changes in neural activity patterns using NMMs. Here, we adapt this latter model by implementing a JR NMM and introducing a feedback mechanism that connects the NMMs’ activity to the production and diffusion of toxic proteins in the AD brain (Fig. 1*A*).

Proteinopathy dynamics are described by the heterodimer model, one of the most common hypotheses that describe the prion-like spreading of toxic proteins. This hypothesis suggests that a healthy (properly folded) protein misfolds when it interacts with a toxic version of itself (misfolded; the prion/seed) following the latter’s structure as a template (Garzón et al., 2021). Therefore, this model includes healthy and toxic versions of amyloid-*β* (*Aβ*, $\tilde{A\beta }$) and tau (*T*, $\tilde{T}$) that are produced, cleared, transformed (from healthy to toxic), and propagated in the SC with N nodes. The proteinopathy dynamics for *i* ∈ [1, *N*] were adapted from Alexandersen et al. (2023) to include the effect of hyperactivity on the enhanced production of *Aβ* (Kamenetz et al., 2003; Cirrito et al., 2005; Stargardt et al., 2015; Yamamoto et al., 2015; Busche and Hyman, 2020) and on the biased prion-like propagation of $\tilde{T}$ to hyperactive regions (Rodriguez et al., 2020); see Table 2 for a description of parameters:

(9) $$\dot{A\beta_{i}}=-\rho \sum_{\;j=1}^{N}L_{ij}\;A\beta_{j}+{\text{\textbackslash{},prod}}_{A\beta }\;q_{i}^{\left(ha\right)}-{\text{clear}}_{A\beta }\;A\beta_{i}-{\text{trans}}_{A\beta }\;A\beta_{i}\;\tilde{A\beta_{i}},$$

(10) $$\dot{\tilde{A\beta_{i}}}=-\rho \sum_{\;j=1}^{N}L_{ij}\;\tilde{A\beta_{j}}-{\text{clear}}_{\tilde{A\beta }}\;\tilde{A\beta_{i}}+{\text{trans}}_{A\beta }\;A\beta_{i}\;\tilde{A\beta_{i}},$$

(11) $$\dot{T_{i}}=-\rho \sum_{\;j=1}^{N}L_{ij}\;T_{j}+{\text{\textbackslash{},prod}}_{T}-{\text{clear}}_{T}\;T_{i}-{\text{trans}}_{T}\;T_{i}\;\tilde{T_{i}}-\text{syn}\;\tilde{A\beta_{i}}\;T_{i}\;\tilde{T_{i}},$$

(12) $$\dot{\tilde{T_{i}}}=-\rho \sum_{\;j=1}^{N}L_{ij}\;\tilde{T_{j}}\;q_{i}^{\left(ha\right)}-{\text{clear}}_{\tilde{T}}\;\tilde{T_{i}}+{\text{trans}}_{T}\;T_{i}\;\tilde{T_{i}}+\text{syn}\;\tilde{A\beta_{i}}\;T_{i}\;\tilde{T_{i}},$$

where prod, clear, and trans stand for rates of protein production, clearance, and transformation from a healthy isoform to a toxic one. syn stands for the synergistic effect between *Aβ* and tau that fosters the conversion of the latter into its toxic isoform (Busche and Hyman, 2020). $q_{i}^{\left(ha\right)}$ represents the effect of hyperactivity on the production of *Aβ* and the propagation of tau (further details below). *ρ* stands for a diffusion constant, and *L*_*ij*_ for the Laplacian diffusion term:

(13) $$L_{ij}=-w_{ij}(t)+\delta_{ij}\sum_{\;j=1}^{N}w_{ij}(t),$$

where *δ*_*ij*_ stands for the Kronecker *δ* (representing the *N* × *N* identity matrix).

**Table 2.** Parameters used in the closed-loop neurotoxicity model

Parameter	Value	Unit	Description
prod_*Aβ*_	3	M ·· · years^−1^	Production rate for *Aβ*
clear_*Aβ*_	3	Years^−1^	Clearance rate for *Aβ*
trans_*Aβ*_	3	M^−1^ ·· · years^−1^	Transformation constant of *Aβ* into its toxic isoform $\tilde{A\beta }$
clearÃβ	2.4	Years^−1^	Clearance rate for $\tilde{A\beta }$
prod_*T*_	3	M ·· · years^−1^	Production rate for *T*
clear_*T*_	3	Years^−1^	Clearance rate for *T*
trans_*T*_	3	M^−1^ ·· · years^−1^	Transformation of tau into its toxic isoform $\tilde{T}$
clearT̃	2.55	Years^−1^	Clearance rate for $\tilde{T}$
syn	0.4	M^−2^ ·· ·years	Synergistic effect between $\tilde{A\beta }$ and $\tilde{T}$
*ρ*	50	cm ·· · years^−1^	Effective diffusion constant
$c_{q}^{\left(\tilde{A\beta }\right)}$	1	M^−1^ ·· · years^−1^	Damage rate for $\tilde{A\beta }$
$c_{q}^{\left(\tilde{T}\right)}$	1	M^−1^ ·· · years^−1^	Damage rate for $\tilde{T}$
$c_{q}^{\left(ha\right)}$	0.01		Damage rate for hyperactivity
$c_{\mathrm{exc}}^{\left(\tilde{A\beta }\right)}$	0.8	Years^−1^	Constant for the effect of $\tilde{A\beta }$ on excitation locally
$c_{inh}^{\left(\tilde{A\beta }\right)}$	0.4	Years^−1^	Constant for the effect of $\tilde{A\beta }$ on inhibition locally
$c_{\mathrm{exc}}^{\left(\tilde{T}\right)}$	1.8	Years^−1^	Constant for the effect of $\tilde{T}$ on excitatory local connectivity
$c_{inh}^{\left(\tilde{T}\right)}$	1.8	Years^−1^	Constant for the effect of $\tilde{T}$ on inhibitory local connectivity
$c_{sc}^{\left(\tilde{T}\right)}$	0.05	Years^−1^	Constant for the effect of $\tilde{T}$ on interregional connectivity
$H_{e_{\max}}$	3.65		Maximum value allowed for *H*_*e*_ parameter change
$C_{ip_{\min}}$	13.25		Minimum value allowed for *C*_*ip*_ parameter change
$C_{ep_{\min}}$	28		Minimum value allowed for *C*_*ep*_ parameter change
$sc_{\mathrm{dam}}$	0.3		Maximum damage of $\tilde{T}$ on interregional connectivity
$q_{\max}^{\left(ha\right)}$	2		Maximum damage value for hyperactivity

*Note*. *Aβ* and *T* stand for healthy amyloid-*β* and tau proteins while $\tilde{A\beta }$ and $\tilde{T}$ stand for their toxic isoforms.

The impact of protein concentration on the BNM is established through two sets of transfer functions. First, a set of damage functions that are used to translate the concentration of toxic proteins into damage variables (Alexandersen et al., 2023):

(14) $${\dot{q}}_{i}^{\left(\tilde{A\beta }\right)}=c_{q}^{\left(\tilde{A\beta }\right)}\;{\tilde{A\beta }}_{i}\;(1-q_{i}^{\left(\tilde{A\beta }\right)}),$$

(15) $${\dot{q}}_{i}^{\left(\tilde{T}\right)}=c_{q}^{\left(\tilde{T}\right)}\;{\tilde{T}}_{i}\;(1-q_{i}^{\left(\tilde{T}\right)}),$$

where $c_{q}^{\left(\tilde{A\beta }\right)}$ and $c_{q}^{\left(\tilde{T}\right)}$ are damage constants for *Aβ* and hp-tau, respectively.

Second, a set of equations that translate the damage just mentioned into NMM parameter changes. We adapted these equations to the JR NMM. It was assumed that each protein ($\tilde{A\beta }$ and $\tilde{T}$) impacts the excitation/inhibition balance in different ways:

∙ $\tilde{A\beta }$ generates neuronal hyperactivity through the impairment of GABAergic synapses (Garcia-Marin, 2009; Limon et al., 2012; Verret et al., 2012; Ulrich, 2015) and through the disruption of glutamate reuptake (Zott et al., 2019).
∙ Soluble hp-tau ($\tilde{T}$) has been shown to change significantly the number and morphology of dendritic spines in pyramidal cells, producing neural silencing that dominates over the $\tilde{A\beta }$ hyperactivation (Merino-Serrais et al., 2013; Busche et al., 2019).

The effects of $\tilde{A\beta }$ will be modeled through changes in the local parameters of the JR NMM including an increase in the amplitude of the excitatory postsynaptic potential (*H*_*e*_) related to the disruption of glutamate reuptake (16) and a reduction of the number of inhibitory synapses to pyramidal cells (*C*_*ip*_) related to GABAergic deficits (Eq. 17). The silencing effects of $\tilde{T}$ will be modeled through a reduction in the number of excitatory interneurons’ synapses to pyramidal cells (*C*_*ep*_), a reduction of interregional weights (*w*_*ij*_), and to *C*_*ip*_.

(16) $$\dot{H_{e_{i}}}=c_{\mathrm{exc}}^{\left(\tilde{A\beta }\right)}\;\;q_{i}^{\left(\tilde{A\beta }\right)}\;(H_{e_{\max}}-H_{e_{i}}),$$

(17) $$\dot{C_{ip_{i}}}=-c_{inh}^{\left(\tilde{A\beta }\right)}\;\;q_{i}^{\left(\tilde{A\beta }\right)}\;(C_{ip_{i}}-C_{ip_{\min}})-c_{inh}^{\left(\tilde{T}\right)}\;\;q_{i}^{\left(\tilde{T}\right)}\;(C_{ip_{i}}-C_{ip_{\min}}),$$

(18) $$\dot{C_{ep_{i}}}=-c_{\mathrm{exc}}^{\left(\tilde{T}\right)}\;\;q_{i}^{\left(\tilde{T}\right)}\;(C_{ep_{i}}-C_{ep_{\min}}),$$

(19) $${\dot{w}}_{ij}=-c_{sc}^{\tilde{T}}\;(q_{i}^{\left(\tilde{T}\right)}+q_{j}^{\left(\tilde{T}\right)})\;(w_{ij}-w_{ij_{\min}}),$$

where

(20) $$w_{ij_{\min}}=w_{ij_{0}}\;(1-sc_{\mathrm{dam}}).$$

$H_{e_{\max}}$, $C_{ip_{\min}}$, $C_{ep_{\min}}$, and $sc_{\mathrm{dam}}$ determine the maximum change allowed per parameter candidate. $c_{\mathrm{exc}}^{\left(\tilde{A\beta }\right)}$, $c_{inh}^{\left(\tilde{A\beta }\right)}$, $c_{\mathrm{exc}}^{\left(\tilde{T}\right)}$, and $c_{inh}^{\left(\tilde{T}\right)}$ stand for constants calibrating the effects of *Aβ* and hp-tau over excitation and inhibition variables.

To this point, we have completed half of the closed-loop model. The second half regards the impact of the simulated neural activity on protein propagation. Neural hyperactivity fosters extracellular secretion and deposition of *Aβ* generating a cyclical phenomenon that reinforces bidirectionally neural hyperactivity and *Aβ* concentration (Kamenetz et al., 2003; Cirrito et al., 2005; Stargardt et al., 2015; Yamamoto et al., 2015; Busche and Hyman, 2020). Additionally, hyperactivity orients the prionic propagation of $\tilde{T}$ in the brain (Rodriguez et al., 2020) following the Braak stages (Braak and Braak, 1991).

To capture these effects in our model, we introduced an additional damage variable for hyperactivity (Eq. 21), based on the averaged increase in firing rate per region compared to baseline:

(21) $${\dot{q}}_{i}^{\left(ha\right)}=c_{q}^{\left(ha\right)}\;(-q_{i}^{\left(ha\right)}+\Delta ha_{i})\;(q_{\max}^{\left(ha\right)}-q_{i}^{\left(ha\right)})\;q_{i}^{\left(ha\right)},$$

where

(22) $$\Delta ha_{i}=ha_{i}/ha_{i_{0}}.$$

The firing rate (*ha*_*i*_) was evaluated as the sigmoidal transformation of incoming afferences to the pyramidal subpopulation (i.e., $S[y_{1_{i}}(t)-y_{2_{i}}(t)]$) and the baseline ($ha_{i_{0}}$) was defined as the regional firing rate measured in a whole brain simulation free from the impact of proteinopathy (Eq. 22). The damage variable $q_{i}^{\left(ha\right)}\in (0,q_{\max}^{\left(ha\right)})$ was implemented as a factor in Equations 9 and 12 multiplying the production of *Aβ* and biasing the Laplacian term for $\tilde{T}$ distribution. When $ha_{i}\approx ha_{i_{0}}$, $q_{i}^{\left(ha\right)}$ tends to one and, therefore, it would not affect the proteinopathy dynamics. $c_{q}^{\left(ha\right)}$ stands for the damage rate for hyperactivity.

### In-silico experiments and simulations

In this work, we performed simulations for several complementary in-silico experiments. First, we explored the parameter spaces of a single JR NMM to evaluate the effects of proteinopathy on neural activity, by varying the AD parameter candidates (*H*_*e*_, *C*_*ip*_, *C*_*ep*_, and *w*_*ij*_). As this first experiment’s simulations were performed with a single node, we accounted for the reduction of interregional connectivity (*w*_*ij*_) produced by hp-tau using the mean of the intrinsic noisy input to the node (*p*), a JR variable that substitutes the interregional afferences not being considered in the simulation. In total, we performed four sets of simulations in this in-silico experiment: the first one focused on the parameters related to *Aβ* effects, and the remaining three focused on parameters related to tau effects. The simulations were performed for 20 s of neural activity discarding the initial 12 to avoid transients, and we recorded information regarding the spectral frequency peak, spectral power in different frequency bands, and firing rate.

Second, we built and simulated the neurotoxicity model to deepen the mechanisms of AD progression. The initial conditions of the proteinopathy dynamics were fixed using a toxic protein seeding following literature on AD staging: the entorhinal cortex for tau (Braak and Braak, 1991) with an initial $\tilde{T}=0.0025$ per region; and insula, precuneus, posterior and isthmus cingulate cortices, and orbitofrontal cortex for *Aβ* (Palmqvist et al., 2017) with an initial $\tilde{A\beta }=0.0125$. Two different integration timelines coexist within the closed-loop model: an integration over the years of AD evolution, and an integration over seconds when simulating the neuronal activity (Fig. 1*C*). The former implies integrating the proteinopathy dynamics each *dt* = 0.25 (years) and evaluating the BNM with the updated parameters due to proteinopathy effects each *dt* = 1 (years). The evaluation of the BNM executes a neural activity simulation for 10 s out of which the initial 2 s are discarded to avoid initial conditions’ transients. Once the BNM is evaluated, the proteinopathy continues to be integrated with updated parameters given the simulated levels of neural activity. We decided to implement two different integration times over years for the proteinopathy and the BNM (i.e., *dt* = 0.25 and *dt* = 1 years, respectively) to reduce the computational load.

We used the neurotoxicity model in several ways: first, we adjusted the model by exploring the impact of the limits of change for the parameter candidates $\left(H_{e_{\max}},C_{ip_{\min}},C_{ep_{\min}},and\;sc_{\mathrm{dam}}\right)$ and two BNM free parameters (*g* and *s*) on the temporal evolution of the model, in terms of averaged frequency peak, relative *α* power, firing rate, and FC. The latter was calculated using the phase locking value (PLV; Lachaux et al., 1999; Bruña et al., 2018) between signals filtered in the *α* band (8–12 Hz):

(23) $${\text{PLV}}_{\;jk}=\frac{1}{T}\left|\sum_{t=1}^{T}e^{i(\phi_{j}(t)-\phi_{k}(t))}\right|,$$

where *ϕ* stands for the instantaneous phase of the signals via the Hilbert transform and *i* for the imaginary unit.

The limits of change for the model parameters determine the extent of the proteinopathy’s impact on JR parameters and, therefore, on the neural activity. We explored those parameter spaces to select a set of values that could reproduce qualitatively the empirical observations reported in the AD continuum: the slowing of *α* frequency, and the rise and decay of relative *α* power, cellular firing rate, and FC. The adjusted parameters are used here as the default values for our model and are included in Tables 1 and 2. Second, we described the outputs of the model with the default parameters including an averaged description of parameter trajectories (Eqs. 16–19), spectra, firing rate, and PLV of the simulated brain regions; third, we disentangled the contribution of *Aβ* and hp-tau to the changes in inhibition by isolating their respective effects on the model evolution. This was achieved by setting to zero alternatively the parameters that define the impact of *Aβ* and hp-tau on inhibition in our model (i.e., $c_{inh}^{\left(A\beta \right)}$ and $c_{inh}^{\left(T\right)}$) and re-computing and comparing the parameter space for the limit of change of *C*_*ip*_ with the original situation. Finally, we used it to evaluate the spatiotemporal evolution of the model with the Braak stages as a reference (see below).

### Spatiotemporal profile assessment

We evaluated the spatial propagation of hp-tau using the Braak stages as a reference and the impact of *Aβ* seeding over the temporal antero-posterior differentiation in terms of firing rate and FC. Braak stages were adapted from Therriault et al. (2022) considering the regions included in our cingulum bundle network (Table 3): *stage one* (*rI*) included the entorhinal cortex; *stage two* (*rII*) included the hippocampus; *stage three* (*rIII*) included the parahippocampus and amygdala; *stage four* (*rIV*) included insula, posterior cingulate, inferior temporal, and inferior parietal; *stage five* (*rV*) included other cortical regions.

**Table 3.** Regions of the cingulum bundle included in the BNM (Bubb et al., 2018)

Left frontal pole	Right frontal pole
Left rostral middle frontal	Right rostral middle frontal
Left caudal middle frontal	Right caudal middle frontal
Left superior frontal	Right superior frontal
Left lateral orbitofrontal^*Aβ*^	Right lateral orbitofrontal^*Aβ*^
Left medial orbitofrontal^*Aβ*^	Right medial orbitofrontal^*Aβ*^
Left insula^*Aβ*; IV^	Right insula^*Aβ*; IV^
Left caudal anterior cingulate	Right caudal anterior cingulate
Left rostral anterior cingulate	Right rostral anterior cingulate
Left posterior cingulate^*Aβ*; IV^	Right posterior cingulate^*Aβ*; IV^
Left isthmus cingulate^*Aβ*^	Right isthmus cingulate^*Aβ*^
Left superior parietal	Right superior parietal
Left inferior parietal^IV^	Right inferior parietal
Left precuneus^*Aβ*^	Right precuneus^*Aβ*^
Left inferior temporal^IV^	Right inferior temporal^IV^
Left parahippocampal^III^	Right parahippocampal^III^
Left hippocampus^II^	Right hippocampus^II^
Left thalamus	Right thalamus
Left amygdala^III^	Right amygdala^III^
Left entorhinal cortex^tau *I*;^	Right entorhinal cortex^tau *I*;^

*Notes*. *Aβ* and tau superscripts denote the regions used as seeding (Braak and Braak, 1991; Palmqvist et al., 2017). I, II, III, and IV superscripts denote the regions included in the Braak stages (Therriault et al., 2022).

We simulated our closed-loop neurotoxicity model by implementing different seeding strategies. The baseline seeding (i.e., fixed strategy) was the same used in previous studies (Thompson et al., 2020; Alexandersen et al., 2023) as stated above. The remaining strategies consisted in randomizing the seeding of *Aβ*, randomizing the seeding of hp-tau, and randomizing both. The randomization respected two conditions: the number of seeded regions had to be the same as in the fixed strategy and the seeding had to be symmetrical between hemispheres. For the antero-posterior differentiation, we also limited the randomization of *Aβ* seeding to posterior areas or anterior areas.

One hundred simulations were performed per seeding strategy. For each timestep, we averaged the concentration of hp-tau in the regions of the Braak stages to determine what Braak regions were presenting higher levels of the protein, and we sorted them into Braak sequences following Putra et al. (2021). Then, we extracted the dominant Braak sequence per simulation as the most repeated sequence in time. For the antero-posterior differentiation, we averaged posterior and anterior neural dynamics and we extracted the time to peak (given the rise and decay in the metrics) of each average.

### Code accessibility

The models described above were implemented in Python 3.9 and executed in a Surface Pro 7 (8Gb RAM) running Windows 11 and a high performance computing cluster. The code used in the paper is freely available online at github.com/jescab01/ADprogress_2023 and as Extended Data in this manuscript.

10.1523/ENEURO.0345-23.2023.d1 Extended Data 1 Code developed in Python 3.9 to simulate and visualize the closed-loop neurotoxicity model. Download Extended Data 1, ZIP file.

## Results

The proposed model for the physiological changes in AD is based on extensive literature that describes the effects of *Aβ* and hp-tau on neural tissue. These changes include the disruption of glutamate reuptake, the reduction of GABAergic synapses, and the reduction of pyramidal dendritic spines. To effectively model these changes, we proposed four parameter candidates that could account for the observed effects. Specifically, we selected two parameters to model the effects of *Aβ* on neural activity including the amplitude of the excitatory postsynaptic potential (*H*_*e*_) to account for the disruption of glutamate reuptake, and the number of local synaptic contacts from inhibitory interneurons to pyramidal neurons (*C*_*ip*_) to account for the reduction of GABAergic synapses. Regarding the effects of tau, we selected three parameter candidates that could effectively model the disruption of pyramidal dendritic spines: *C*_*ip*_ mentioned above, *C*_*ep*_ as the local synaptic contacts from excitatory interneurons to pyramidal neurons, and *w*_*ij*_ as the interregional SC weights.

### Single-node experiments favor inhibition over excitation to explain hyperactivity

In order to gain a better understanding of the impact of toxic proteins on neural behavior, we explored the parameter spaces of the proposed candidates (*H*_*e*_, *C*_*ip*_, *C*_*ep*_, and *w*_*ij*_) through single-node NMM simulations. Note that we used the mean of the intrinsic noisy input to the node (*p*) to account for the reduction of *w*_*ij*_ as simulations were performed with a single node.

**Figure 2.**
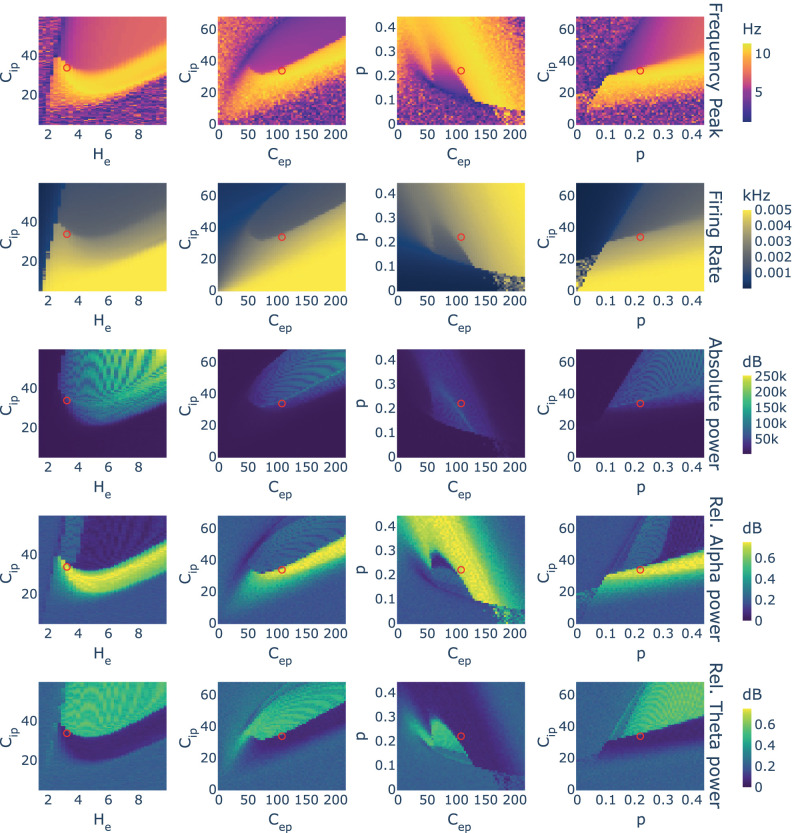
Single-node in-silico experiment. The behavior of a single NMM was simulated varying parameter values. Four different experiments are presented in columns exploring different combinations of parameters. In rows, measures derived from the simulations: oscillatory frequency peak of the node, mean firing rate, absolute power, and the relative power in two frequency bands: *α* and *θ*. The red circle points out the default parameter values. A 2D representation of each parameter change is shown in Extended Data Figure 2-1. Sample time series for the different oscillatory regimes are shown in Extended Data Figure 2-2.

In general terms, and in line with previous analysis of the JR model (Grimbert and Faugeras, 2006; Spiegler et al., 2010), results showed two main patterns of oscillation: a fast limit cycle in the *α* frequency band (8–12 Hz) and a slow limit cycle in *θ* frequency band (4–8 Hz). These two behaviors were categorically separated in the parameter spaces as can be observed in the frequency and relative power charts of Figure 2. The noisy regions surrounding the main limit cycles correspond to the fixed point states of JR that are found at both extremes of the excitation–inhibition continuum. In these states, the model might not have enough activation to give rise to an oscillatory behavior, or it might be saturated by a too-high excitation. In both states, the noise acts as a driver giving rise to low-amplitude and low-frequency noisy oscillations, and the more stable the fixed point (i.e., more extreme excitation–inhibition balance) the lower the frequency and amplitude. Accordingly, note that the noisy regions coincide with the low-power regions in the absolute power plots of Figure 2.

10.1523/ENEURO.0345-23.2023.f2-1 Figure 2-1 2D representations of single node experiments varying only one parameter at a time (i.e., H_e_, C_ip_, C_ep_, p). Dashed lines showing the default parameter value. Markers' colour scales are the same as in the heatmaps of Figure 2. Download Figure 2-1, TIF file.

10.1523/ENEURO.0345-23.2023.f2-2 Figure 2-2 Timeseries and spectra for three different regimes shown in Figure 2: slow limit cycle, fast limit cycle and fixed point state. Two traces from independent simulations are shown. Download Figure 2-2, TIF file.

Focusing on the limit cycle regions, the parameter candidates related to intranode excitation (i.e., *H*_*e*_ and *C*_*ep*_; see first and second–third columns of Fig. 2, respectively) showed quadratic relationships in their parameter spaces, producing curved isopleth borders separating the slow and fast oscillatory behaviors (i.e., *θ* and *α*, respectively). These curves represented a convex relationship between the parameters and the neural behavior in terms of frequency, power, and firing rate. The position of their original values derived from biology (Jansen and Rit, 1995) differed with respect to the vertex of the quadratic curve: the original *H*_*e*_ was found at lower values than the vertex, while *C*_*ep*_ was found at higher values.

For the other two parameters (i.e., *C*_*ip*_ and *p*), the observed changes inside the limit cycle regions were monotonical, and better described as logistic relationships in which the changes happen quickly at the edge between behaviors (Extended Data Fig. 2-1). These relationships were direct in the case of p with both frequency peak and firing rate, in contrast to the inverse relationship found for *C*_*ip*_ (i.e., lower inhibition raised the firing rate and the frequency of oscillation). Note that lowering inhibition out of the limit cycle regions had two different effects on firing rate and spectra. The firing rate kept rising monotonically as inhibition lowered, while the frequency peak and power tended to be reduced getting into the noisy low amplitude and slow region commented above.

In this model, two parameters could explain the hyperactivation produced by *Aβ*: *C*_*ip*_ lowering down due to the reduction of GABAergic synapses and *H*_*e*_ rising due to the disruption of glutamate reuptake. Assuming that the original values of these parameters, which were based on biological observations (Jansen and Rit, 1995), are a good approximation of the underlying ground truth, our experiment would favor a hypothesis in which the hyperactivity found in AD is explained more likely by the reduction of *C*_*ip*_ than by the increase of *H*_*e*_ given that the latter would induce an initial reduction of activation levels that is not expected in AD (see the effects of lowering *C*_*ip*_ and rising *H*_*e*_ in Fig. 2, second row, first column).

### Closed-loop neurotoxicity model: from proteins to neural activity

To explore the link between proteinopathy and neural activity, we extended a previously published multiscale model of AD evolution (Thompson et al., 2020; Alexandersen et al., 2023) by implementing a biologically plausible NMM (Jansen and Rit, 1995) and establishing a bidirectional interaction between neural activity (Eqs. 1–6) and proteinopathy (Eqs. 9–12). The proteinopathy starts from an initial distribution of toxic seeds, and during the temporal evolution of the model, the BNM integrates the effects of those proteins over neural tissue, which in turn influences the production of *Aβ* and the propagation of hp-tau based on firing rate.

**Figure 3.**
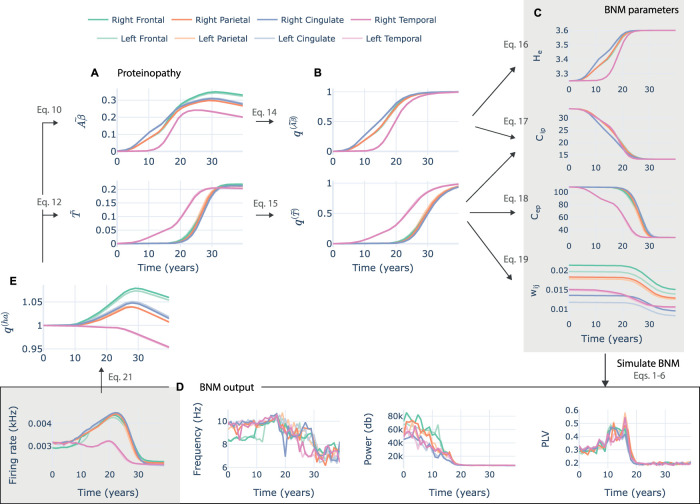
Evolution of the parameters, state variables, and neural activity outputs from the closed-loop neurotoxicity model. Results are averaged over regions included in eight categories including frontal, parietal, temporal, and cingulate regions. ***A***, Concentration of toxic proteins over time during model execution. ***B***, Damage variables that determine the impact of the proteinopathy over the neural activity (i.e., JR-BNM parameters). ***C***, JR-BNM parameters change over time. ***D***, Functional measures derived from the simulation of the BNM including spectral frequency peak and power, PLV, and firing rate. The latter is used in the calculation of *q*^(*ha*)^. ***E***, Damage variable *q*^(*ha*)^ that determines the impact of hyperactivity on the production and propagation of toxic proteins. The average of the parameter changes shown in (***C***) are plotted over the single-node experiments’ heatmaps in Figure 3-1.

The regional evolution of the proteinopathy follows a sigmoidal shape with a final slight decay in *Aβ* toxic concentration (see average curves in Fig. 3*A*). The model captures the time delays between the rise of *Aβ* and hp-tau reported in the literature (Therriault et al., 2022). The effect of proteinopathy on neural activity was modeled through changes in the NMM parameters mediated by a damage variable (Fig. 3*B*). These changes were monotonical and followed the sigmoidal shape of the proteinopathy and its damage. The impact of *Aβ* over the neural tissue was modeled as a reduction of *C*_*ip*_ and an increase in *H*_*e*_, while the impact of hp-tau was modeled as reductions of the local and interregional connectivity parameters explored before (*C*_*ip*_, *C*_*ep*_, *w*_*ij*_) as shown in Figure 3*C* and Extended Data Figure 3-1. The changes in parameters due to the proteinopathy generated a rise and decay in firing rate over time (Fig. 3*D*) that affected the production of *Aβ* and the distribution of hp-tau through a hyperactivity damage variable (Fig. 3*E*).

10.1523/ENEURO.0345-23.2023.f3-1 Figure 3-1 Parameter trajectories of the simulated closed-loop model with default parameters on heatmaps from single node experiments. Trajectories are represented as curves with varying colours. The colour represents time: starting in red and ending with blue. Note that heatmaps are extracted from single-node simulations in which just two parameters are varied at a time, while the trajectories imply a 4-dimensional parameter change. Therefore, the underlying heatmaps should be interpreted just as an orientation of what might happen when changing parameters in one direction. Download Figure 3-1, TIF file.

**Figure 4.**
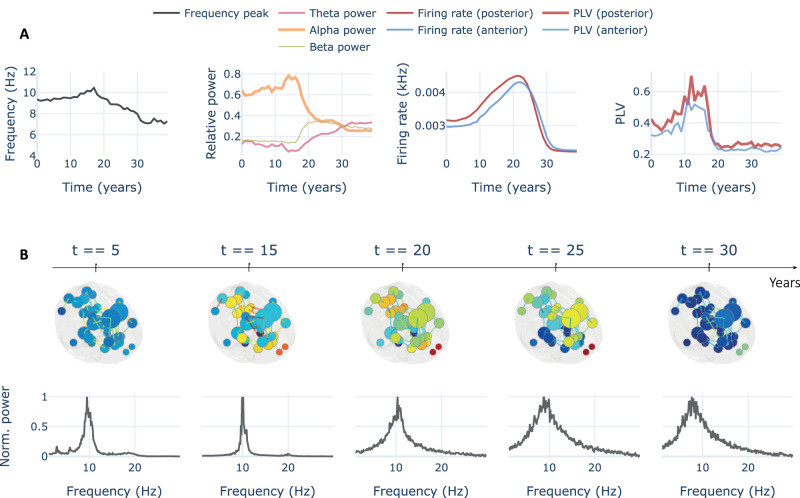
Functional measures from the closed-loop neurotoxicity model. Metrics averaged from the simulation shown in Figure 3. ***A***, We extracted spectral frequency peak, relative band power, firing rate, and *α* FC from the simulation over 40 years of the neurotoxicity model. A quadratic relationship is found over time for all these metrics (i.e., measures increase early in time and decrease later). ***B***, Temporal excerpts of the same simulation including 3D representations of the BNM with node size as degree, node color as firing rate (orange/red is a higher rate), and edge colors as PLV. Last row shows averaged normalized spectra for all the regions included in the model. FC evolution in other frequency bands is shown in Figure 4-1. Regional spectra at several time points are shown in Figure 4-2.

The specific limits of change of the parameters (included in Table 2) were fitted by iterative explorations of the parameter spaces generated with the neurotoxicity model to achieve the reproduction of some of the main pathophysiological changes of AD evolution (Maestú et al., 2021): frequency slowing, reduction in relative *α* power, hypo-/hyperactivity, and hypo-/hypersynchrony in *α* (Fig. 4). We noticed that the reduction of *α* FC does not translate into an enhancement of FC in lower frequency bands (i.e., *θ*/*δ*; Extended Data Fig. 4-1). Additionally, we could not reproduce a clear temporal distinction between antero-posterior effects of the proteinopathy on FC (i.e., posterior regions are affected first) as it has been previously proposed (Fig. 4*A*, PLV). However, we also found slight antero-posterior differences in magnitude between those regions for FC and firing rate.

10.1523/ENEURO.0345-23.2023.f4-1 Figure 4-1 Averaged FC (PLV) of the simulated closed-loop model with default parameters for different frequency bands: delta (2 - 4 Hz), theta (4 - 8 Hz) and alpha (8 - 12 Hz). Download Figure 4-1, TIF file.

10.1523/ENEURO.0345-23.2023.f4-2 Figure 4-2 Normalized spectra per region over time (yrs.). Note the transition of all spectra to more noisy states with lower frequency peaks. Download Figure 4-2, TIF file.

### Hyperactivity is not directly linked to hypersynchrony

The parameter space explorations that allowed us to fit the model are used in the following to establish relationships between different variables and outcomes derived from the neurotoxic model simulation. To obtain these parameter space explorations, we simulated the model with different values of the parameter candidates’ limits of change.

**Figure 5.**
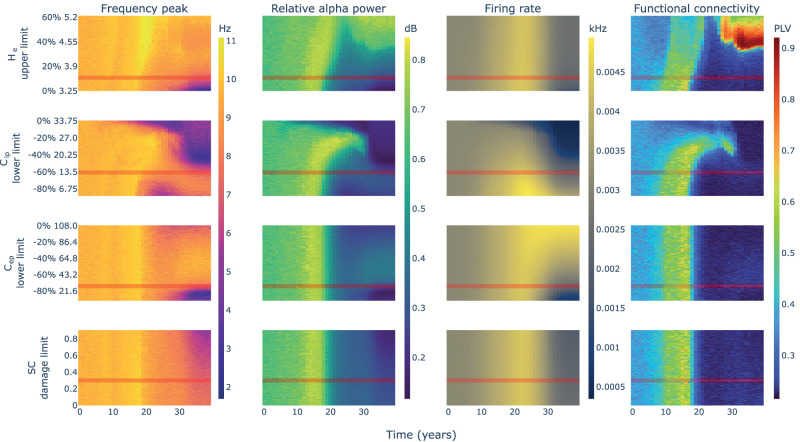
Parameter spaces for the limits of change of the JR-BNM parameter candidates. For each parameter candidate (rows) and limit value explored (heatmaps *y*-axis), one simulation of 40 years (heatmaps *x*-axis) is performed. For each simulation, four neural activity measures are extracted including averaged frequency peak, relative *α* power, firing rate, and FC (columns). When exploring a parameter candidate, all other parameters are kept fixed with their default values that are highlighted in red and included in Table 2. These default values were used in the simulations for Figures 3 and 4. Note how taking apart from zero the limits of change impact differently the resulting behavior of the model. Two additional parameter spaces were computed to define values for *g* and *s* (Fig. 5-1).

The model behavior could be analyzed by dividing it into two stages: a first stage in which we find an increase in relative *α* power, firing rate, and FC; and a second stage in which all these measures decay (Fig. 5). These two stages are not synchronized for all measures. We observed a delayed peak in firing rate at the time interval of years 18–25, while all other measures peak earlier in time between years 10 and 18. From year 18 to 25, the firing rate keeps rising while power and FC have already lowered. This observation suggests a dissociation between firing rate and FC that has not been previously established.

10.1523/ENEURO.0345-23.2023.f5-1 Figure 5-1 Parameter spaces to select working point. Selected parameters were g=25 and s=20 m/s (see red highlights). Note how the rising of g leads to a situation in which no FC rise is observed, similar to the reduction of s, due to high early FC levels. Also, lowering g leads to the prebifurcation regime of the JR NMMs, a situation in which the spectral frequency peak lowers towards the delta band. Download Figure 5-1, TIF file.

### The changes in inhibition lead the evolution of AD

Two parameter candidates could be responsible for the initial rising stage: the increase of *H*_*e*_ and the reduction of *C*_*ip*_. Looking at the parameter spaces, only the changes to the *C*_*ip*_ limits showed a disruption in the rising phase when reduced less than a 20% (Fig. 5, second row). On the other hand, changes to the limits of *H*_*e*_ did not show any significant effect in this stage, even using an upper limit equal to 0% (i.e., keeping the parameter fixed in time) the rise in all measures could still be observed (Fig. 5, first row). This would suggest that the rising stage is mainly controlled in our model by the changes in inhibition (i.e., the reduction of *C*_*ip*_ parameter) representing the *Aβ* dependent reduction of GABAergic synapses.

The decaying stage required a more complex analysis. In the previously exposed single-node experiments, we observed that lowering inhibition showed a transition from the slow limit cycle, to the fast limit cycle, to the noisy low frequency and amplitude oscillation in which excitation–inhibition imbalance saturates the regional response and leads the JR NMMs to operate in a fixed point state (Fig. 2 and further explanations above). In that context, the reduction of *C*_*ip*_ itself could explain the decay stage for frequency peak and relative *α* power by entering the noisy region where lower inhibition (i.e., further imbalance of excitation–inhibition) leads to a more stable JR fixed point state and lower spectral frequencies. Interestingly, those spectral changes were time-locked with the changes in *α* band FC (Figs. 4 and 5). Despite explaining the decay stage for those three measures, the reduction of *C*_*ip*_ could not explain the decay for firing rate. Lowering inhibition produced a monotonic ascent as could be expected from Figure 2 even in the noisy low-amplitude region of the single-node parameter spaces.

The decay of firing rate could then be explained by another two parameter candidates related to the tau-derived reduction of pyramidal dendritic spines: the reduction of *C*_*ep*_ and the reduction of SC weights. The parameter spaces showed that only the reduction of local excitation (i.e., *C*_*ep*_) interacted with the firing rate (Fig. 5, third column). Disabling the change for *C*_*ep*_ (i.e., *C*_*ep*_ change limit of 0%) showed a plateau in firing rate during the decay stage, while activating its reduction showed a progressive effect on the decay of the firing rate (Fig. 5, third row, compare −40%, −60%, and −80% change limits). This effect was not found for different levels of damage to the SC weights.

### Amyloid-*β* controls changes in inhibition

From the analysis above, we can derive that the disruption of inhibition is a relevant driver of AD progress. Given that both *Aβ* and hp-tau contribute to the disruption of inhibition by reducing GABAergic synapses and reducing dendritic spines, respectively, we wondered whether they contribute differently to the inhibitory-related effects reported above.

**Figure 6.**
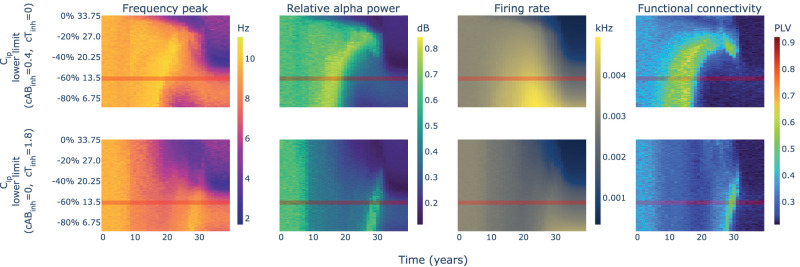
Parameter spaces for *C*_*ip*_ isolating the effects of *Aβ* and hp-tau. First row shows the results of simulating the neurotoxicity model with different limits of change for *C*_*ip*_ isolating the effects of *Aβ* on inhibition, while the second row shows the results of simulating the same parameter space isolating the effects of hp-tau on inhibition.

To disentangle their contribution, we computed an additional set of parameter spaces in which we evaluated independently the impact of the inhibitory disruption caused by each protein (Fig. 6). We observed a clear similarity between the parameter space of *Aβ*-only effects and the previously shown full model in Figure 5. The isolated effect of tau could also generate a slight rise and decay in power, frequency, and FC delayed from the *Aβ* effect and shorter in time, with a more moderate change in firing rate. This would suggest that both proteins could produce to some extent the effects observed in AD due to the disruption of inhibition, although with different timings and hyperactivity levels.

### Toxic seeding determines the spatiotemporal profile of the evolution

The spatiotemporal sequence of hp-tau spreading in AD has been accurately characterized through the Braak stages. We used this characteristic spatiotemporal sequence to evaluate the spatial accuracy of the tauopathy simulated in our model, and the impact of the selected toxic proteins’ seeding regions on its spatial evolution. We implemented four seeding strategies including fixed seeding, *Aβ* randomized, hp-tau randomized, and both randomized. We performed 100 simulations per strategy and we measured the concentration of hp-tau in the regions of the Braak stages extracting the dominant spatial sequence of propagation per simulation.

**Figure 7.**
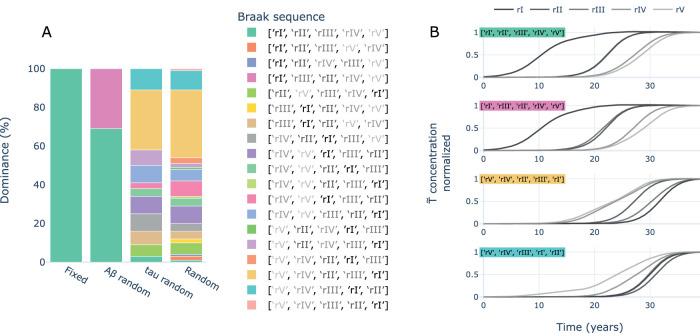
Dominance of Braak sequences per seeding strategy. ***A***, The percentage of simulations per seeding mode in which different Braak sequences of hp-tau propagation were dominant. Fixed implied seeding a usual, *Aβ* random implied randomizing *Aβ* seeding and using the fixed version for hp-tau, vice-versa for tau random, and random implied randomizing both *Aβ* and hp-tau seeding. ***B***, Samples of the most representative Braak sequences showing the normalized concentration of $\tilde{T}$ over time.

Results showed that fixed (i.e., as usual) and *Aβ* random seeding strategies reproduced effectively the Braak staging in 100% and 70% of the simulations, respectively (Fig. 7*A*). In these simulations, just a slight temporal shift separated stages II (i.e., hippocampus) and III (i.e., parahippocampus and amygdala), and this way 30% of the simulations with *Aβ*-random seeding showed a sequence in which hp-tau reached stage III before stage II. Interestingly, randomizing tau seeding produced a variety of Braak sequences that lowered the dominance of the theoretical sequence to less than 5% of the simulations, at the same levels of randomizing both seeding locations. Examples of these sequences in time are shown in Figure 7*B*. These observations posit the importance of the localization of hp-tau seeds (i.e., early accumulations) to determine the evolution of its propagation.

In addition, we wondered to what extent the antero-posterior temporal differentiation of the neurophysiological changes observed along the AD continuum could be related to the seeding of *Aβ* and hp-tau. We performed an additional set of simulations randomizing the seeding of *Aβ* and hp-tau and measuring the differences in firing rate and FC between anterior and posterior regions.

**Figure 8.**
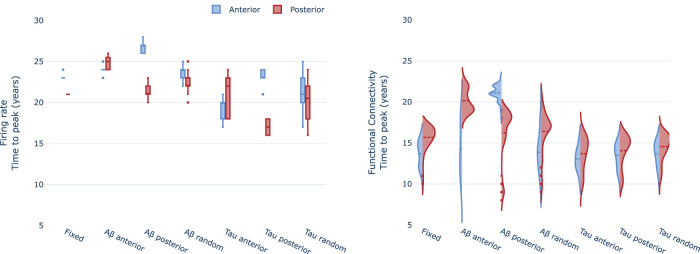
Impact of *Aβ* and hp-tau seeding on the antero-posterior temporal differentiation for firing rate (top) and FC (PLV; bottom). The neurotoxicity model shows a rise and decay for several variables including firing rate and FC. We measured the time elapsed to that peak for anterior and posterior regions as a proxy to understand where the neurophysiological changes happened before. Samples of the temporal evolution for each seeding strategy and for both firing rate and FC are represented in Figure 8-1.

We observed a stable precession of posterior regions in terms of firing rate for the cases of fixed seeding (i.e., following Palmqvist et al., 2017), randomizing *Aβ* and hp-tau in posterior regions, and randomization them over all regions (Fig. 8). Only in the case of anterior seeding, the model showed a slightly faster peak in anterior regions. This suggests that hyperactivity tends to appear earlier in posterior regions disregarding where *Aβ* and hp-tau start to accumulate. Note that *Aβ* and hp-tau seedings affected differently the levels of hyperactivity shown by the regions: non-*Aβ*-seeded and tau-seeded regions lowered their maximum firing rates as shown in Extended Data Figure 8-1.

10.1523/ENEURO.0345-23.2023.f8-1 Figure 8-1 Samples of the evolution for firing rate (left column) and FC (i.e., PLV; right column) in the antero-posterior differentiation experiments. In rows, each of the four seeding implemented strategies. The effect on FC is limited to a temporal shift of the curves, however, the seeding affects the level of hyperactivity reached by the anterior or posterior regions. Download Figure 8-1, TIF file.

In contrast, the antero-posterior temporal differentiation for FC relied more on the seeding of *Aβ*. When the seeding was located in posterior regions they peaked earlier while, on the contrary, when anterior regions were seeded they tended to peak earlier. Interestingly, these two strategies generated a general delay in the time to peak both in the seeded region and in the complementary. Note that changing hp-tau seeding reduced the time to peak but did not affect the antero-posterior differentiation relative to the fixed strategy.

## Discussion

In this study, we developed a multiscale closed-loop neurotoxicity model of AD integrating a BNM that reproduced brain activity with another modeling the production and propagation of toxic proteins in the brain, both influencing each other through biologically plausible links. Our results showed that the disruption of inhibition was favored to explain cellular hyperactivity, and indeed that inhibition was key to explaining the AD-related changes in frequency, power, and FC. These changes in inhibition were primarily attributed to *Aβ* effects. Additionally, our results suggested a disconnection between cellular hyperactivity and interregional hypersynchrony. Finally, we observed a significant association between the spatiotemporal profile of the evolution of AD to the initial localization of the accumulation of AD proteins.

Single-node experiments favored the lowering of inhibition (related to GABAergic synapses’ disruption) over the rising of excitation (related to impaired glutamate reuptake) to explain the characteristic hyperactivity produced by toxic *Aβ* on neural tissue. This idea is in line with previous animal studies showing a direct association between the levels of GABAergic synaptic activity and the hyperactivity found in the vicinity of amyloid plaques (Busche et al., 2008, 2015). The conclusions derived from the single-node experiments are based on the assumption that the original definition of the JR parameters approximates well the characteristics of neural populations. Another definition of parameters could lead to different results. However, note that JR parameters were derived from empirical studies, for instance, *C*_*ip*_ and *C*_*ep*_ were suggested in light of histological research establishing proportions of synaptic connections between different cell types in several neocortical regions (Jansen and Rit, 1995). In any case, these experiments propose testable predictions regarding the extent to which these two *Aβ*-derived physiopathological phenomena may contribute to neural activation in AD.

Additionally, single-node experiments showed different effects for the changes implemented to the parameter candidates in terms of neural activity measures. Inside the limit cycle space, we observed quadratic relationships with firing rate and power for the local excitation parameters (i.e., *H*_*e*_ and *C*_*ep*_), while a monotonical relationship was found for the inhibitory parameter (i.e., *C*_*ip*_; Fig. 2). These observations suggest two different roles for excitation and inhibition. While inhibition would predispose the system to order neural activation into synchronized oscillations, excitation is needed to trigger this mechanism. Therefore, both increasing and decreasing excitation levels could lead to a rise in cellular firing rate: while low excitation may become ineffective to trigger inhibition, high excitation could overcome the capacity of inhibition to organize action potentials and generate oscillatory behavior. This type of contradictory effect (i.e., rising excitation parameter could lower firing rate, and vice-versa) has been reported in empirical studies under the name of inhibitory stabilization of network dynamics, in which, for instance, the stimulation of inhibitory neurons produce a counterintuitive suppression of inhibitory firing (Sadeh and Clopath, 2020; Sanzeni et al., 2020). This mechanism is characteristic of networks with strong recurrent connectivity and protects the system from unstable dynamics such as epileptic seizures.

In agreement with single-node experiments, the simulation of the multiscale neurotoxicity model gave a prominent role to inhibition leading the neural activity changes typically observed in AD. Despite both *Aβ* and hp-tau can be associated with changes in inhibition—due to the disruption of GABAergic synapses, and to the reduction of dendritic spines that reduces the number of inhibitory inputs to pyramidal cells, respectively—in the model, *Aβ* was linked to the main inhibitory changes that lead AD evolution. However, it was also shown that hp-tau alone could generate inhibitory effects in the same direction but weaker, shorter, and delayed in time.

The relationship between cellular hyperactivity and interregional hypersynchrony in AD has been suggested (Sepulcre et al., 2017; Koelewijn et al., 2019; Maestú et al., 2021; Stam et al., 2023) but not directly tested to our knowledge. The rationale behind this association is based on the studies linking *Aβ* independently with both hyperactivity and hypersynchrony. On one hand, some studies report higher cellular activation, or lower firing thresholds, in regions affected by *Aβ* (Busche et al., 2008; Palop and Mucke, 2010). On the other hand, some studies report spatial correlations between *Aβ* accumulation (and hp-tau later) and the levels of FC, which are mainly observed within the default mode network (Mormino et al., 2011; Schultz et al., 2017; Quevenco et al., 2020; Ranasinghe et al., 2020; Schoonhoven et al., 2023). From these observations, it has been derived that *Aβ* produces cellular hyperactivity, and that cellular hyperactivity, in turn, produces FC hypersynchrony. In our study, we show that hyperactivity and hypersynchrony might not be directly linked, as we found a reduction of FC while *Aβ* accumulation was still enhancing cellular hyperactivity, suggesting a temporal mismatch between the two. In contrast, FC levels were strongly associated in time with changes in spectral power. We believe that this dissociation between spectral power and firing rate is essential to understand the hyperactivity/hyperconnectivity question. Whenever hyperactivity overcomes the capacity of inhibition to order neural firing into an oscillatory behavior, the regional activity may become noisier and therefore more difficult to synchronize interregionally. However, when hyperactivity moves the excitation/inhibition ratio toward a more balanced state, larger oscillations appear that may give rise to FC changes in a network.

The simulation of the closed-loop neurotoxicity model showed a sigmoidal shape in the evolution of the concentration of toxic proteins with a final slight decay for $\tilde{A\beta }$ that is found in the empirical literature (Therriault et al., 2022). This decay is associated in our model with the reduction of hyperactivity that lowers the production of *Aβ* and therefore leads to the recovery of the production-clearance balance. The impact of hyperactivity on the production of *Aβ* has been suggested in previous studies and linked to alterations in the endocytic machinery that could increase the rate of clathrin-mediated endocytosis of the amyloid precursor protein (APP) and also enlarge APP endosomes (Stargardt et al., 2015). After releasing these APP vesicles, a molecular cascade mediated by *β*-secretase and *γ*-secretase cleaves the molecule to generate *Aβ* (Haass and Selkoe, 2007).

In this work, we used the entorhinal cortex as the seeding region for hp-tau propagation. However, recent studies suggest that the classical Braak staging of tau pathology in AD (Braak and Braak, 1991) may not offer a comprehensive view and may disregard alternative tau epicenters and propagation trajectories (Franzmeier et al., 2020; Lamontagne-Kam et al., 2023). From the epicenters, hp-tau spreads to functionally connected regions (Franzmeier et al., 2022; Schoonhoven et al., 2023) producing different network disruptions and clinical profiles. In a study by Vogel et al. (2021), they identified four distinct tau propagation patterns, each capable of explaining between 18 and 33% of the cases. We believe that our framework poses a good opportunity to advance the understanding and prediction of personalized tau propagation trajectories. Our approach considers not only structural and FC networks but also accounts for the progressive changes in neural activity due to proteinopathy.

An aspect that has not been captured by our model is the effect of chronic neuroinflammation. It may affect the normal functioning of glial cells, disrupting the clearance function of both microglia and astrocytes and, therefore, fostering protein accumulation and neuroinflammation (Heneka et al., 2015; Henstridge et al., 2019). Although it is not the only additional aspect that could be implemented in the model due to its impact on AD progression (e.g., vascular damage leading to reduced protein clearance, infections such as herpes virus, genetic factors, etc.), we expect that by including these glial effects, we could reproduce the slight decay in hp-tau concentration that is found in the literature, but not reproduced in our results. Further modeling studies including additional AD-related factors are needed.

Regarding simulated neural activity, our model qualitatively reproduced some of the most relevant biomarkers along the AD continuum, including the slowing of the *α* frequency, the rise and decay of relative *α* power, firing rate, and *α* FC, and the Braak stages (Maestú et al., 2021). However, we could not directly reproduce two FC-related phenomena reported in the literature: the rise of FC for lower frequency bands (Nakamura et al., 2017; Schoonhoven et al., 2022), and the spatiotemporal dissociation in FC between posterior and anterior brain regions (López-Sanz et al., 2016; Nakamura et al., 2017, 2018; Pusil et al., 2019). The latter was dependent on spatial seeding of *Aβ* that, by including the orbitofrontal cortex in addition to the precuneus and posterior cingulate cortex (Palmqvist et al., 2017), balanced antero-posterior changes in time. We believe that assuming this spatial seeding to be correct, the result could be expected empirically. However, we wonder whether the technical difficulties in detecting accumulations of *Aβ* could hinder the detection of earlier accumulations in posterior regions that could explain the electrophysiological evidence mentioned above.

Previous modeling research has explored the mechanisms underlying frequency slowing in AD, including the work by Alexandersen et al. (2023) that served as the basis for the research presented here. They found that the oscillatory changes in the AD continuum were more related to local neurodegeneration than to the disruption of interregional SC. Our results reproduced most of their observations, supporting the local neurodegeneration hypothesis. Additionally, van Nifterick et al. (2022) presented an exploration of six excitatory-inhibitory mechanisms that could lead to the slowing of AD. They found that both hyperexcitation and disinhibition (related to the *Aβ* neurotoxic effects) could lead to oscillatory slowing. Our model integrated three of these mechanisms: rise of the excitatory postsynaptic potential (*H*_*e*_), lowering of the inhibitory coupling (*C*_*ip*_), and lowering of excitatory coupling (*w*_*ij*_). Their results fit into the single-node experiments parameter spaces presented here, which offer a comprehensive picture in which non-linear relationships between the levels of excitation–inhibition and the spectral frequency changes can be observed. Finally, other studies have previously modeled the frequency slowing observed in AD by manipulating the time constants of the NMMs. Ranasinghe et al. (2022) fitted the model parameters to match empirical spectra from patients, finding relationships between *Aβ* accumulation and inhibitory time constants changes, and between hp-tau accumulation and excitatory time constants. Also, Stefanovski et al. (2019) modeled the inhibitory disruption through changes in the inhibitory time constants linked to *Aβ* accumulation. These models point to inhibitory disruptions due to *Aβ* accumulation, in line with the results presented here. We did not include effects on time constants in our modeling approach considering that the empirical evidence for AD at the cellular level has reported more often changes in the magnitude of excitation/inhibition instead of changes in neural time constants.

This work provides a comprehensive and self-contained mechanistic explanation of how different factors contribute interactively to the course of AD and, therefore, it becomes relevant for urgent clinical needs. First, the observed hyperexcitation is the treatment target of the approved symptomatic drug Memantine (Reisberg et al., 2003, 2006) and other treatments currently under investigation such as the anti-epileptic drug Levetiracetam (Vossel et al., 2021). Several clinical trials in AD have determined that selecting the correct individuals at the correct disease stage for a particular intervention is crucial, our model aims to help in this process in the future. Furthermore, both increased neural activity and altered synchrony can be targeted with invasive or non-invasive brain stimulation (Chang et al., 2018). Deep brain stimulation in the fornix has positively affected cognition, but mainly if the stimulation site precisely activates memory networks (Ríos et al., 2022). The promising effects of transcranial magnetic stimulation depend on a particular adaptation between the used frequencies and their effects on the excitation–inhibition balance (Weiler et al., 2019). Neuromodulation cannot only improve but also deteriorate the function of a diseased brain, and the prediction of an individual stimulation regime requires a deep understanding of the underlying mechanisms. The same applies to pharmacological treatments: while the recent success of Lecanemab in slowing down cognitive decline is promising and raises hopes for the amyloid hypothesis after decades (van Dyck et al., 2023), the observed effects are minimal, and the long-term outcome is still unclear. One avenue toward more efficient use of potentially disease-modifying treatments is, again, the choice of the correct timing for the therapy. The closed-loop model in this work can even explain several potential reasons that hinder an effective slowing of the neurodegenerative process: it explains how both *Aβ* and hp-tau can drive the vicious circle independent from each other, and it formalizes how the self-sustaining process evolves even without the presence of the initiating factor. Altogether, only a holistic understanding of AD will open the possibility to once even preventing the development of dementia, and computational neuroscience can powerfully contribute to this.

## Synthesis

Reviewing Editor: Arvind Kumar, KTH Royal Institute of Technology Decisions are customarily a result of the Reviewing Editor and the peer reviewers coming together and discussing their recommendations until a consensus is reached. When revisions are invited, a fact-based synthesis statement explaining their decision and outlining what is needed to prepare a revision will be listed below. The following reviewer(s) agreed to reveal their identity: Ana P Vidal.

Synthesis

In this paper authors have presented a computational model of progression of Alzheimer's disease. The model suggests that hyperexcitability does not always imply hypersynchrony. They provide new results about how functional connectivity may evolve and provide an explanation of frequency slowing. Overall it is an interesting paper and improves our understanding of AD. The two reviewers however have raised some major concerns that need to be addressed before we can consider this manuscript for publication. Some of the key points are as follows:

One of the main results about frequency slowing is not really obvious from the results. It seems that frequency slowing is in fact frequency switching from alpha to theta band in some regions

The reviewers also noted that some additional results should be included in the manuscript eg. spatial inhomogeneity of the activit, PLV for theta band activity. There are other similar suggestions in the reviewers' comments

The methods and in particular the models should be described in a more clear way. The reviewers have made several suggestions in this regard. Please refer to their comments below

Finally the work should be properly placed in comparison to the previous work. Reviewers' would like to see a comparison with previous work. Also some references should be appropriately cited.

In the following I have appended the reviewers' feedback. Please consider that in your revision and provide a point-by-point reply.

Reviewer #1

This paper builds on the work of Alexandersen (2023), where a model for Alzheimer's disease progression is formulated. In this paper, the model has been modified to include the Jansen-Rit model, which is a biophysically-flavored model of neuronal activity. The results from Alexandersen (2023) are reproduced alongside novel results on functional connectivity and amyloid seeding. Parameters of a single model are also explored to investigate the spectral properties of the JR model.

The paper is well-written and the results are interesting. However, in my opinion, the paper requires re-editing and possible work before publication. I have split my comments into major comments and minor comments. Please do not be intimidated by the number of minor comments, they are minor and should be quick and easy to amend. I hope you will find my comments useful.

MAJOR COMMENTS

Major comment 1: Frequency slowing

The authors state that the model captures the observed frequency slowing seen in the alpha frequencies in MEG/EEG of Alzheimer's patients. However, as has been demonstrated in the paper, there is an abrupt transition from alpha to theta oscillations in the single-node JR model. In Figure 3, the frequency peak and normalized power are averages of all connectome regions. It is my impression that the frequency peak in Figure 3 appears to slow down because some regions are transitioning to theta oscillations, not because the alpha frequency slows down. If this is the case, the model does not capture alpha peak frequency slowing, but rather the relative increase in theta power.

If the authors can demonstrate that the alpha peak itself is slowing in individual regions, that would be ideal. If not, the manuscript should reflect the fact that alpha peak slowing is not observed, at least in this sense. (this is not a bad thing, to my knowledge no model can yet realistically capture dynamics in multiple frequency bands simultaneously)

Major comment 2: PLV in the theta band

The PLV in the theta band should have been included. Either the authors can reproduce the results together with PLV bandpassed in the theta/delta range, or it should be made clear that the disassociation between PLV and firing rate may be different for PLV in the theta range.

Major comment 3: Discussion in relation to previous work

The results of the paper are not discussed in relation to results from previous work on computational modeling of Alzheimer's disease. The results of previous work should be compared and, in particular, the mechanisms proposed to explain hyperactivity/slowing.

As the model presented herein is an extension of the model introduced by Alexandersen (2023), the results herein should be compared.

Nifterick (2022) should be compared.

As the authors use a JR model similar to Stefanovski (2019), these results should be compared as well.

Ranasinghe (2022) should also be compared.

Major comment 4: Introduction and earlier work

From the introduction, it is not clear that this work builds on the Alzheimer's disease model introduced by Alexandersen (2023). This is made clear in the Methods and Results, but should be clear from reading the introduction as well. Lines 385-389 could be moved to the Introduction, for example (or something similar)

MINOR COMMENTS

Minor comment 1:

In the abstract, the model is described as "unique" and in the significance statement the paper is described as "groundbreaking". It is my opinion these descriptions should be removed, as the abstract and significance statement itself should rather state why it is unique and groundbreaking, not state that it is.

Minor comment 2:

Lines 52-53 lack citation (GABAergic synapses)

Minor comment 3:

Lines 88-89, not all neural mass models are second-order ODes.

Minor comment 4:

The presentation of the mathematical model in Section 2.3 can be made clearer. Equations (8) and (9) should be grouped together. There should be (in each line or at the bottom) a definition of "i" (the indexing), for example, i=1,...N. In Line 170, there seems to be global noise mean and std but in the equations they are indexed; this should be clarified. The equations in Section 2.4 are very long due to words being used as opposed to constants. I understand that this is practical, but as these constants rarely come up in the text I think it would be better to use symbols. The equations are too large to fit on the page, and symbols like TAU are easily confused when they are differentiated (single dot over u or over the whole thing?). In equation (18) a minus sign is missing, I believe. There should be a "where" between equations (19) and (20). Sometimes parameters are explained after a set of equations sometimes they are not, I would prefer a short explanation each time (and refer to the tables for an overview). In equations (21) and (22), there should be a "where" between them, and variables/parameters should be described briefly.

Minor comment 5:

Lines 235-237 should be made clearer. In lines 237-239, the assumptions are not motivated yet.

Minor comment 6:

In lines 245-247, the "mean input to node p" should be clarified, I am not 100% sure what it means.

Minor comment 7:

Lines 260-264 are not clear. This is an important part of the model and should be made clearer to the reader.

Minor comment 8:

Line 337: which previous analysis? Citations?

Minor comment 9:

In line 356, what does "convex relationship" and "logistic relationship" mean? If it is the curves in parameter space, I think it suffices to refer to the figure.

Minor comment 10:

It would be nice with a refresher on how the parameters from the 1995 JR paper were derived. Are they reasonable to use here? This should be a key point in the Discussion.

Minor comment 11:

Figure 2C is not too clear, what does the grey line represent?

Minor comment 12:

Lines 450-455 should be in the single-node section (this actually summarizes that section well and would be neat to have as a conclusion there)

Minor comment 13:

Line 460, the functional connectivity changes and alpha spectral power occur simultaneously because the PLI uses signal bandpassed in the alpha frequency. This should be explained to the reader.

Minor comment 14:

Just of potential interest to authors: Figure 6 is similar to work done by Prama Putra on simulating Braak staging patterns with spreading models and might be useful:

Putra P, Thompson TB, Chaggar P, Goriely A. Braiding Braak and Braak: Staging patterns and model selection in network neurodegeneration. Netw Neurosci. 2021 Nov 30;5(4):929-956. doi: 10.1162/netn_a_00208. PMID: 35024537; PMCID: PMC8746141.

Minor comment 15:

The term closed-loop is not clarified (it can have many meanings). If it means that the model lacks external input, then I do not think that it is necessary to describe the model as closed-loop. In my experience, most whole-brain models are. Closed-loop can be removed from the text, as I think it is more confusing than clarifying, but this is up to the authors.

Minor comment 16:

lines 542-544, I do not think Figure 5 shows enough evidence to say that tau produces all these effects in isolation. From Figure 5, it does not seem like tau alone can cause hyperactivity. This is not mentioned in the text, I believe. I read it as both tau and amyloid-beta alone can reproduce all the effects but from Figure 5 only some effects are explained. It would be good to make this clear to the reader.

Minor comment 17:

Lines 555-559, this is a big assumption. The parameter space of JR is huge, and it should be noted that different parameterizations could produce different results. The parameterization of JR from 1995 should be defended to support the claims of the paper.

Minot comment 18:

In the significance statement, "contrary to prior assumptions" does the authors mean "contrary to prior beliefs"? I do not mean to be pedantic, but it can be confused with modeling assumptions.

Reviewer #2

In this paper, the authors present a comprehensive, multiscale mathematical framework aimed at elucidating the progression of Alzheimer's disease (AD). The authors introduce a model that combines prion-like protein propagation to simulate the spreading of abnormal tau and amyloid-beta, with a neural mass model for assessing functional activity. The authors put forth a sound mathematical framework that is effectively expounded upon within the manuscript with an extensive analysis of the emerging dynamics in relation to AD pathology. Their main findings, in particular the discovery of a causal disconnection between hyperactivity and hypersynchrony in the model, are well-supported by the analyses. These findings contribute valuable insights to the trending discussion in the literature regarding the role of hyperactivity in AD pathology. As such, I am pleased to recommend the publication of this paper in eNeuro.

I do have some minor suggestions that I believe can enhance the presentation and readability of the model and the results. While the manuscript is eloquently composed and nicely written, the intricate nature of the model and analysis set-up, featuring various model components, parameters, and analytical tests, can be challenging for readers to navigate. This is especially relevant for the broad readership of eNeuro, which may not uniformly have an extensive mathematical background. The changes I propose below (in the order in which they appear in the manuscript) are intended to clarify and emphasize the main aspects of the manuscript:

1. Some discussion on the reasoning for selecting this particular structural network is needed. Why restrict the network to the cingulum bundle? Is this choice supported by the literature? Moreover, what is the expected impact on the model's findings? I would also appreciate including an image of the SC matrix (alongside the schematic figure I suggest below).

2. The format of the equations causes readability issues, and the format is somewhat inconsistent throughout the manuscript:

1. I suggest avoiding the use of "cdots" to indicate scalar multiplication and to drop the temporal depdencies. Both these things appear in the first set of equations (1-6, JR model) and are slowly dropped in subsequent systems. I suggest they are removed altogether for the sake of simplicity. If necessary, cdots might be substituted by small spaces to clearly separate variables.

2. In all equation systems, particularly eqs. 1-6 and eqs. 9-12, please use consistent justification to aid readability (i.e. all equations with the same justification so that equal symbols align consistently).

3. Continuing with the first set of equations (1-6, JR model), I strongly recommend expressing the system as three 2nd order differential equations rather than six 1st order equations (as it appears now), as the former is generally more readable. Furthermore, please use square brackets in eqs. 5,6 at the largest level, instead of parentheses as it is written now. Similarly, the variable input(t) is missing the subscript i. In order to improve readability, it may also be an option to substitute y0,y1,y2 for x,y,z to eliminate the subindex and avoid double subscripts. Adopting more descriptive variable names, such as pn (pynamidal neurons), ei (exc. Interneurons) or ii (inhibitory interneurons) is also an option the authors may consider. Similarly, please consider changing "input" with a simpler variable name such as "I" (see also following comment).

4. For the second set of equations (9-12, protein dynamics), readability is also hindered, particularly due to the protracted variable names. While I understand this is done so that the variables are more descriptive, in this case it hinders the readability of the equations. Hence, I strongly recommend employing simple, short variable names. For instance, use the symbol "\tau" instead of "TAU", and add a bar to the toxic variables (\bar{a\beta} and \bar{\tau}) instead of adding the termination "t". The current notation has led to typos and inconsistencies in eqs. 11 and 12, with the use of both "T" and "\tau" instead of "TAU", and in eq. 12 "TAU_t" is used instead of "TAUt". Please also simplify the notation for "clear", "trans" etc. (at the very least using non-italic fonts for spelled out words might be sufficient -this also applied to "input" in the previous set of equations- but I strongly recommend simplifying the notation here). Finally, some variable definitions are missing: the variable "syn" is not defined below that I can see, and pa brief definition of q^ha and a reference to its further discussion would be helpful to avoid unnecessary confusion.

5. Similarly, in eqs. 14 and 15 a shorter name for "A\beta t _{damrate}" would enhance clarity.

6. In general the parameters introduced in each equation remain undefined. While in the first set of equations there is a reference to a table describing the parameters, this referencing is less evident in the subsequent equation sets. Hence, I suggest including brief definitions of the main parameters, or a more explicit reference to the corresponding table.

7. Can \Delta ha_i (eq. 22) become negative in later stages? How is this handled in the model?

3. Given the complexity of the modeling frameworks, I suggest including an initial figure with a schematic overview of the modeling framework (which may also include the SC matrix as suggested above). I suggest two panels, one for the JR model, illustrating the coupling within and between regions, and a second panel describing the different components of the BNM model and their interactions (i.e. in the same spirit for the central panel in fig. 2, but more detailed).

4. In line 218-219, please briefly define "He", "Cip", "Cep", as these are only defined before on a table and not the main text.

5. I am curious for the choice of temporal scales for the two models (line 260). Why is the NMM not run between each iteration of the neurotoxicity model? Have you explored the effect of the choice of timescales?

6. Please briefly defined "g" and "a" in line 267, since these have not been defined in the main text.

7. The final paragraph of section 2.5 is hard to follow. I found the expression "adjusting the model" rather unclear, and moreover the analyses described in this section were not apparent to me until after reading the results. From the results section I understood that the exploration of the effect of the extreme values for the different variables serves to characterize their effect on the functional dynamics, and to select "adjusted parameters" that best describe AD pathology. I do not understand however how are these adjusted parameters selected? Are these adjusted parameters used in figures 3 and 6?

8. Line 289-290, on the amyloid-beta seeding profiles, should be the first line of the subsequent paragraph. Moreover, the antero-posterior differentiation is not properly introduced in the introduction of the manuscript.

9. In section 3.1, it would be helpful to show some representative time series of the NMM variables for each of the three regimes.

10. The first discussion of the effect of model parameters on the phase diagrams shown in figure 1 involves He and Cep, however, this combination is not shown in the figure, which is counter-intuitive. I understand the reasoning for showing He and Cip instead, but perhaps the authors can edit the text or the figure to circumvent this issue.

11. In figure 1, I suggest the authors to delineate the fast and slow limit-cycle regions with some contour lines to aid readability. I am also curious for why three different colormaps are used in this figure.

12. In lines 369-371 it is mentioned that "the firing rate kept rising monotonically as inhibition lowered, while the frequency peak and power tended to be reduced" however I find it difficult to conclude this from the colormaps alone. A supplementary figure showing the 1D curves resulting from varying one parameter only (Cip in this case), corresponding for instance to the red point marked in the figure, would suffice to address this issue.

13. In lines 391-392 it says that "the BNM integrates the effects of those proteins over neural tissue, which in turn influences the production of Abeta based on firing rate." Doesn't neural activity affect tau spreading as well?

14. In figure 2, the authors show the evolution of different variables of the neurotoxicity model over time, averaged over brain regions. However different regions have different temporal profiles, hence I think it would be useful to show here some individual curves for different regions, in order to illustrate the spatial heterogeneity. Or does this figure correspond to the dynamics simulated only for one region? Could the authors clarify this point? In any case, a figure illustrating the spatial heterogeneity would be a valuable addition to the manuscript in my opinion.

15. Also in figure 2, I am really not a fan of the style used to show the different scales in panel C. I would suggest plotting normalized values (from 0 to 1) and indicating the minimum and maximum values for each varible in the caption. Otherwise, changing the grid on the y-axis so that the ytick-labels correspond to more extreme values, and perhaps adding also a vertical arrow for each of the variable scales, can help readability.

16. In the result section 3.2 (and in general throughout the results) it would help to refer back to the relevant equations from the methods section. Also please use the same notation as in the methods, e.g. in figure 2B and 2E. I also think there is a typo in figure 2C such that "Cie" and "Cee" should respectively be "Cip" and "Cep".

17. The effect of the limits of the parameters (figure 4) is discussed clearly in section 3.4. However, it is also mentioned in sections 3.2 (paragraph starting on line 408) and 3.3 (starting line 423). Rather than helpful, I found this repetition confusing. Furthermore, figure 4 is currently mentioned before figure 3, and it is not clear whether the results of figure 4 affect figure 3 (i.e. to what parameter configuration does figure 3 correspond to)? How were the values of the parameters "adjusted"? I think the manuscript would benefit from a more clear separation between the different sections, by i.e. removing the aforementioned paragraphs, and including in section 3.4 or the corresponding methods section some more detail on how the parameters were adjusted. It is not clear to me whether that would require inverting the order of some result sections and/of figures 3 and 4, but in any case a separate section discussing the parameter "adjusting" analysis and the phase diagrams in figure 4 would be helpful. Similarly, an intuitive explanation of the effect of the limits of the model parameters should be included already in the corresponding methods section.

18. I recommend that the authors consider the inclusion of an additional schematic figure at the end of section 3.4. This figure could serve as a concise visual summary of the primary findings from the analyses, particularly highlighting which parameters can lead to each of the distinct Alzheimer's disease pathologies under consideration, according to the model.

19. Why is the study done in figure 7 performed for amyloid beta and not tau?

20. I found a few spelling errors and typos:

1. Line 55: the reference "Maestú et al." is missing parentheses.

2. "TVB" in line 151, which I assume stands for the virtual brain, is not defined

3. In line 170, please write "standard deviation" instead of "std".

4. There seems to be an extra "\newline" inserted before some of the equations (eq. 1, eq. 9, eq. 14). Please remove this.

5. The phase variable "phi" in eq. 23 is not defined.

6. In line 269, I believe it should say "functional connectivity" instead of "PLV"?

21. The literature review is extensive, but I recommend considering also the following studies:

1. Regarding tau spreading:

1. Schoonhoven, Deborah N., et al. "Tau protein spreads through functionally connected neurons in Alzheimer's disease: a combined MEG/PET study." Brain (2023): awad189.

2. Lamontagne-Kam, Daniel, et al. "Implication of tau propagation on neurodegeneration in Alzheimer's disease." Frontiers in Neuroscience 17 (2023).

2. Regarding the link between network hyperexcitability / hyperactivity and network hypersynchrony:

1. Stam, C. J., et al. "Network Hyperexcitability in Early Alzheimer's Disease: Is Functional Connectivity a Potential Biomarker?." Brain Topography (2023): 1-18.

## Author Response

Dear reviewers, Thank you very much for your time, effort, and your insightful comments on the manuscript "A multiscale closed-loop neurotoxicity model of Alzheimer's disease progression explains functional connectivity alterations". We believe that your observations have contributed greatly to enhance the quality, readability, and consistency of our work. We have discussed and implemented most of your observations in the manuscript.

In addition to reviewers' observations, we have detected and corrected a mismatch between the mathematical definition of hyperactivity damage in the manuscript and its implementation in Python. This affected equation 21 and implied updating the parameter value HAdamrate=0.01 in order to obtain similar hyperactivity damage values as in the previous formulation. The new equation 21 calculates the derivative for hyperactivity damage as:

With this new formulation, we avoid previous instabilities by limiting the damage value with the expression . Additionally, we included a dampening element in , that allows the damage to tend to baseline when the increase in hyperactivity comes back to its own baseline at 1 (i.e., ha(t) = ha0).

Based on these changes, we recomputed all simulations to check that the new definitions did not affect the results, not finding any significant differences. We updated all figures in the manuscript with new simulated data, and the figures within this rebuttal are computed with the new mathematical definitions.

Reviewer #1 This paper builds on the work of Alexandersen (2023), where a model for Alzheimer's disease progression is formulated. In this paper, the model has been modified to include the Jansen-Rit model, which is a biophysically-flavoured model of neuronal activity. The results from Alexandersen (2023) are reproduced alongside novel results on functional connectivity and amyloid seeding. Parameters of a single model are also explored to investigate the spectral properties of the JR model.

The paper is well-written and the results are interesting. However, in my opinion, the paper requires re-editing and possible work before publication. I have split my comments into major comments and minor comments. Please do not be intimidated by the number of minor comments, they are minor and should be quick and easy to amend. I hope you will find my comments useful.

MAJOR COMMENTS Major comment 1: Frequency slowing The authors state that the model captures the observed frequency slowing seen in the alpha frequencies in MEG/EEG of Alzheimer's patients. However, as has been demonstrated in the paper, there is an abrupt transition from alpha to theta oscillations in the single-node JR model. In Figure 3, the frequency peak and normalized power are averages of all connectome regions. It is my impression that the frequency peak in Figure 3 appears to slow down because some regions are transitioning to theta oscillations, not because the alpha frequency slows down. If this is the case, the model does not capture alpha peak frequency slowing, but rather the relative increase in theta power.

If the authors can demonstrate that the alpha peak itself is slowing in individual regions, that would be ideal. If not, the manuscript should reflect the fact that alpha peak slowing is not observed, at least in this sense. (this is not a bad thing, to my knowledge no model can yet realistically capture dynamics in multiple frequency bands simultaneously) Thank you very much for the comment, it is really interesting. We agree that there are abrupt transitions from alpha to theta oscillations in single node experiments. However, if we take into consideration the direction of change of the parameters involved (see Figure 1 below), we could argue that those abrupt changes might not be involved in the evolution of the closed-loop model.

The parameters (i.e., He, Cip, Cep, and SC weights) are changing linked to the evolution of the proteinopathy, in which toxic AB concentration rises first (before hp-tau) influencing parameters He (reduction of the glutamate reuptake) and Cip (reduction of GABAergic synapses). In Figure 1, we plotted the trajectories of parameters He and Cip in a default simulation of the closed-loop model over the heatmaps from single node experiments. As shown in the figure, those trajectories are not expected to cross the abrupt transition towards the slow oscillation limit cycle in JR, but to move further apart from that border in the direction of the low power and noisy region described in the text.

The only parameter spaces in which we see a border crossing are in the third column of figure 1, representing the parameter spaces for Cep and p/weights. These parameters change later in time as they are related to hp-tau accumulation (note the trajectory colours indicating time), implying that previous changes to other parameters (i.e., Cip and He) might have reshaped the parameter space and therefore that heatmap would not be so informative.

Figure 1. Parameter trajectories of the simulated closed-loop model with default parameters on heatmaps from single node experiments. Trajectories are represented as curves with varying colour. The colour represents time: starting in red and ending with blue. Note that heatmaps are extracted from single-node simulations in which just two parameters are varied at a time, while the trajectories imply a 4-dimensional parameter change. Therefore, the underlying heatmaps should be interpreted just as an orientation of what might happen changing parameters in one direction.

Given this analysis, we do not expect the spectral transitions to be abrupt, but we expect all the nodes to transition smoothly towards a situation of low power, relatively higher levels of noise, and slower frequency peaks (as described in sections 3.1 and 3.4 of the manuscript). This situation corresponds to a fixed-point state of the JR model powered up by noise.

To confirm these ideas, we have extracted additional plots from the simulated closed-loop model including regional spectra at different points in time. Figure 2 shows the normalized spectra per region at five equally distant timepoints (i.e., yrs = [0, 8, 16, 24, 32]). We normalized the spectra such that the maximal power was equal to 1 for a better visualization of the frequency peak transition. Normalized spectra show that:

- at time 0, most of the regions (38/40) are oscillating in the alpha regime, only the frontal pole shows a slower oscillation related to the slow limit cycle of the JR described in the manuscript in lines 337-342. These slower oscillators are likely related to lower structural connectivity degree in those regions reducing their baseline inputs.

- At year 8, some of the alpha regions accelerate their peaks.

- At year 16, some of alpha regions that previously had accelerated, now they decelerate (e.g., lh-insula, lh-lateralorbitofrontal, rh-posteriorcingulate) still in alpha band. Other oscillators that did not accelerate before, accelerate now (e.g., rh-rostralanteriorcingulate, lh-inferiorparietal, lh-caudalmiddlefrontal). The frontal poles switched their dynamics to the alpha limit cycle.

- From year 24, the spectra appear noisier and frequency peaks tend to low down.

Figure 2. Normalized spectra per region over time (yrs.). Note the transition of all spectra to more noisy states with lower frequency peaks.

This figure demonstrates that individual nodes are experiencing the spectral changes over time and, in particular, that they are experiencing the slowing of alpha peak. We believe that the fixed-point state of JR to which the model tends corresponds to the second bifurcation of the JR, in which the excitation-inhibition imbalance (high excitation) saturates the oscillatory behaviour of the system.

These plots have been included in the manuscript as extended data for figures 1 and 3, respectively.

Major comment 2: PLV in the theta band The PLV in the theta band should have been included. Either the authors can reproduce the results together with PLV band passed in the theta/delta range, or it should be made clear that the disassociation between PLV and firing rate may be different for PLV in the theta range.

Thank you very much again for this sharp comment. Indeed, plotting PLV values filtered in other frequency bands would enhance the interpretability of the results. Figure 3 shows the evolution of PLV for delta (2 - 4 Hz), theta (4 - 8 Hz) and alpha (8 - 12 Hz) bands. PLV in all these frequency bands seem to be stable at year 20 after a decay phase. Only alpha band shows an initial increase in FC and a posterior decay. Ideally, the loss in alpha FC is translated into a rise of theta FC as reported in literature (Nakamura et al., 2017; Schoonhoven et al., 2022), unfortunately this is not captured by our model.

This plot will be included as extended figure for article's figure 3, and the issue was commented in the results section (lines 434-436) and discussion (lines 688-690).

Figure 3. Averaged functional connectivity (PLV) along the simulated years for different frequency bands: delta (2 - 4 Hz), theta (4 - 8 Hz) and alpha (8 - 12 Hz) bands.

Major comment 3: Discussion in relation to previous work The results of the paper are not discussed in relation to results from previous work on computational modeling of Alzheimer's disease. The results of previous work should be compared and, in particular, the mechanisms proposed to explain hyperactivity/slowing.

- As the model presented herein is an extension of the model introduced by Alexandersen (2023), the results herein should be compared.

- Nifterick (2022) should be compared.

- As the authors use a JR model similar to Stefanovski (2019), these results should be compared as well.

- Ranasinghe (2022) should also be compared.

Thank you for the comment. You are right, a discussion regarding previous work should be included in the manuscript. We will add the following paragraph to the discussion section (lines 701-729): "Previous modelling research has explored the mechanisms underlying frequency slowing in AD, including the work by Alexandersen et al. (2023) that served as the basis for the research presented here. They found that the oscillatory changes in the AD continuum were more related to local neurodegeneration than to the disruption of interregional structural connectivity. Our results reproduced most of their observations, supporting the local neurodegeneration hypothesis. Additionally, Van Nifterick et al. (2022) presented an exploration of six excitatory-inhibitory mechanisms that could lead to the slowing of AD. They found that both hyperexcitation and disinhibition (related to the AB neurotoxic effects) could lead to oscillatory slowing. Our model integrated three of these mechanisms: rise of the excitatory postsynaptic potential (He), lowering of the inhibitory coupling (Cip), and lowering of excitatory coupling (wij). Their results fit into the single node experiments parameter spaces presented here, which offer a comprehensive picture in which non-linear relationships between the levels of excitation-inhibition and the spectral frequency changes can be observed. Finally, other studies have previously modelled the frequency slowing observed in AD by manipulating the time constants of the NMMs. Ranasinghe et al. (2022) fitted the model parameters to match empirical spectra from patients, finding relationships between AB accumulation and inhibitory time constants changes, and between hp-tau accumulation and excitatory time constants. Also, Stefanovski et al. (2019) modelled the inhibitory disruption through changes in the inhibitory time constants linked to AB accumulation. These models point to inhibitory disruptions due to AB accumulation, in line with the results here presented. We did not include effects on time constants in our modelling approach considering that the empirical evidence for AD at the cellular level has reported more often changes in the magnitude of excitation/inhibition instead of changes in neural time constants." Major comment 4: Introduction and earlier work From the introduction, it is not clear that this work builds on the Alzheimer's disease model introduced by Alexandersen (2023). This is made clear in the Methods and Results but should be clear from reading the introduction as well. Lines 385-389 could be moved to the Introduction, for example (or something similar) We made clearer the foundations of the work in the last paragraph of the introduction by including a reference to the work of Alexandersen et al. (2023) (lines 99-101): "In this study, we extended a previously published multiscale model of AD evolution based on neuroimaging data that includes molecular, cellular, and interregional features of the brain (Alexandersen et al., 2023)." MINOR COMMENTS Minor comment 1:

In the abstract, the model is described as "unique" and in the significance statement the paper is described as "groundbreaking". It is my opinion these descriptions should be removed, as the abstract and significance statement itself should rather state why it is unique and groundbreaking, not state that it is.

We totally agree with you. Those words were removed. Thank you.

Minor comment 2:

Lines 52-53 lack citation (GABAergic synapses) You are right. We were leaning on the review by Maestú et al. 2021. We added more specific citations in those lines.

Minor comment 3:

Lines 88-89, not all neural mass models are second-order ODes.

Completely right. Changed to sets of differential equations. Thank you.

Minor comment 4:

The presentation of the mathematical model in Section 2.3 can be made clearer: • Equations (8) and (9) should be grouped together.

Done. • There should be (in each line or at the bottom) a definition of "i" (the indexing), for example, i=1,...N.

Included in line 157. • In Line 170, there seems to be global noise mean and std but in the equations they are indexed; this should be clarified.

Gaussian noise parameters p (mean) and η (standard deviation) are defined globally, but they are implemented as a local and independent noise per node. This means that although p will be shared by all nodes, η will vary in time and between nodes. Therefore, we will use more consistently p without indexing and ηi(t) to indicate the local and time varying implementation of the noise. • The equations in Section 2.4 are very long due to words being used as opposed to constants. I understand that this is practical, but as these constants rarely come up in the text, I think it would be better to use symbols. The equations are too large to fit on the page, and symbols like TAU are easily confused when they are differentiated (single dot over u or over the whole thing?).

We have replaced "TAU" variable with "T", and using ~ to refer to toxic isoforms. Now, equations fit into the page. We would prefer keeping the prod, clear and trans variables to facilitate readability of readers with non-mathematical background. • In equation (18) a minus sign is missing, I believe.

Nicely spotted! Thank you very much. • There should be a "where" between equations (19) and (20).

Certainly, thank you! • Sometimes parameters are explained after a set of equations sometimes they are not, I would prefer a short explanation each time (and refer to the tables for an overview).

Remaining parameters have been explained after the equations. • In equations (21) and (22), there should be a "where" between them, and variables/parameters should be described briefly.

Included.

Minor comment 5: • Lines 235-237 should be made clearer.

You are right, the explanation was not comprehensive. We have updated it by stating how we extracted the firing rate from simulations: "To capture these effects in our model, we introduced an additional damage variable for hyperactivity (eq. 21). It was based on the averaged increase in firing rate per region compared to baseline, considering baseline a whole brain simulation free from the impact of proteinopathy (eq.22). The firing rate (hai) was evaluated as the sigmoidal transformation of pyramidal subpopulation's inputs (i.e., S[y1(t) - y2(t)])." • In lines 237-239, the assumptions are not motivated yet.

If we understood correctly, the motivation for the implementation of those elements into the equations was stated above that paragraph: "Neural hyperactivity fosters extracellular secretion and deposition of Aβ generating a cyclical phenomenon that reinforces bidirectionally neural hyperactivity and Aβ concentration (Cirrito et al., 2005; 229 Yamamoto et al., 2015; Kamenetz et al., 2003; Busche and Hyman, 2020; Stargardt et al., 2015). Additionally, hyperactivity orients the prionic propagation of TAUt in the brain (Rodriguez et al., 2020) following the Braak stages (Braak and Braak, 1991)." Minor comment 6:

In lines 245-247, the "mean input to node p" should be clarified, I am not 100% sure what it means.

We introduced a further description of the JR parameter "p": "As simulations were performed with a single node, we accounted for the reduction of interregional connectivity (wij) produced by hp-tau using the mean of the intrinsic noisy input to the node (p), a JR variable that substitutes the interregional afferences not being considered in the simulation." Minor comment 7:

Lines 260-264 are not clear. This is an important part of the model and should be made clearer to the reader.

We rephrased and extended those lines to make them clearer: "Two different integration timelines coexist within the closed-loop model: an integration over the years of AD evolution, and an integration over seconds when simulating the neuronal activity. The former implies integrating the proteinopathy dynamics each dt=0.25 (yrs) and evaluating the BNM with the updated parameters due to proteinopathy effects each dt=1 (yrs). The evaluation of the BNM executes a neural activity simulation for 10 seconds out of which the initial 2 seconds are discarded to avoid initial conditions related transients. Once the BNM is evaluated, the proteinopathy continues to be integrated with updated parameters given the simulated levels of neural activity. We decided to implement two different integration times over years for the proteinopathy and the BNM (i.e., dt=0.25 and dt=1 yrs., respectively) to reduce the computational load derived from simulating the BNM. " Minor comment 8:

Line 337: which previous analysis? Citations? Completely right. Citations added.

Minor comment 9:

In line 356, what does "convex relationship" and "logistic relationship" mean? If it is the curves in parameter space, I think it suffices to refer to the figure.

We believe that these terminology helps describing the parameter spaces, and we would prefer to keep them.

Minor comment 10:

It would be nice with a refresher on how the parameters from the 1995 JR paper were derived. Are they reasonable to use here? This should be a key point in the Discussion.

We have added a short reminder of the parameter definition in JR to discussion section.

Minor comment 11:

Figure 2C is not too clear, what does the grey line represent? The gray line represents the averaged decay in structural connectivity weights along years.

Minor comment 12:

Lines 450-455 should be in the single-node section (this actually summarizes that section well and would be neat to have as a conclusion there) Thanks for the comment. Actually, in single node experiments, we have not yet shown the outputs of the closed-loop model, and hence, we haven't presented parameter trajectories yet. We believe that it could be misleading to talk about parameter trajectories in that section.

Minor comment 13:

Line 460, the functional connectivity changes and alpha spectral power occur simultaneously because the PLI uses signal bandpassed in the alpha frequency. This should be explained to the reader.

Thank you, we added a specific mention to the bandpass filter in that sentence.

Minor comment 14:

Just of potential interest to authors: Figure 6 is similar to work done by Prama Putra on simulating Braak staging patterns with spreading models and might be useful:

Putra P, Thompson TB, Chaggar P, Goriely A. Braiding Braak and Braak: Staging patterns and model selection in network neurodegeneration. Netw Neurosci. 2021 Nov 30;5(4):929-956. doi: 10.1162/netn_a_00208. PMID: 35024537; PMCID: PMC8746141.

Indeed, we used their work as an inspiration for that part. We missed including it in methods, and we should have certainly done it. We have included the citation in methods section.

Minor comment 15:

The term closed-loop is not clarified (it can have many meanings). If it means that the model lacks external input, then I do not think that it is necessary to describe the model as closed-loop. In my experience, most whole-brain models are. Closed-loop can be removed from the text, as I think it is more confusing than clarifying, but this is up to the authors.

We called the model closed-loop as it closes the AD cyclical interaction between protein accumulation and hyperactivity in both directions. We included a figure in the methods section describing the modeling framework used in the manuscript. The use of the "closed-loop" expression was explained in the caption.

Minor comment 16: lines 542-544, I do not think Figure 5 shows enough evidence to say that tau produces all these effects in isolation. From Figure 5, it does not seem like tau alone can cause hyperactivity. This is not mentioned in the text, I believe. I read it as both tau and amyloid-beta alone can reproduce all the effects but from Figure 5 only some effects are explained. It would be good to make this clear to the reader.

We agree with the observation. We will remove from the sentence the part regarding tau effects. Additionally, this affects the abstract in which the same claim is included. We rephrased the abstract to focus on the effects of AB.

Minor comment 17:

Lines 555-559, this is a big assumption. The parameter space of JR is huge, and it should be noted that different parameterizations could produce different results. The parameterization of JR from 1995 should be defended to support the claims of the paper.

We have rewritten the paragraph to defend the biological plausibility of parameters in JR.

Minot comment 18:

In the significance statement, "contrary to prior assumptions" does the authors mean "contrary to prior beliefs"? I do not mean to be pedantic, but it can be confused with modeling assumptions.

Completely right, we changed the word "assumptions" with "beliefs". Thank you very much.

Reviewer #2 In this paper, the authors present a comprehensive, multiscale mathematical framework aimed at elucidating the progression of Alzheimer's disease (AD). The authors introduce a model that combines prion-like protein propagation to simulate the spreading of abnormal tau and amyloid-beta, with a neural mass model for assessing functional activity. The authors put forth a sound mathematical framework that is effectively expounded upon within the manuscript with an extensive analysis of the emerging dynamics in relation to AD pathology. Their main findings, in particular the discovery of a causal disconnection between hyperactivity and hypersynchrony in the model, are well-supported by the analyses. These findings contribute valuable insights to the trending discussion in the literature regarding the role of hyperactivity in AD pathology. As such, I am pleased to recommend the publication of this paper in eNeuro.

I do have some minor suggestions that I believe can enhance the presentation and readability of the model and the results. While the manuscript is eloquently composed and nicely written, the intricate nature of the model and analysis set-up, featuring various model components, parameters, and analytical tests, can be challenging for readers to navigate. This is especially relevant for the broad readership of eNeuro, which may not uniformly have an extensive mathematical background. The changes I propose below (in the order in which they appear in the manuscript) are intended to clarify and emphasize the main aspects of the manuscript:

1. Some discussion on the reasoning for selecting this particular structural network is needed. Why restrict the network to the cingulum bundle? Is this choice supported by the literature? Moreover, what is the expected impact on the model's findings? I would also appreciate including an image of the SC matrix (alongside the schematic figure I suggest below).

Thank you very much for the comment. We decided to use a subset of regions for the structural network in order to reduce the computational load of the BNMs simulations. We selected the CB as it is defined as an anatomical network that overlaps with many of the default mode network (DMN) connections (Bubb et al., 2018). And we were interested in the DMN because we were modeling resting-state M/EEG data in which the default DMN activates prominently (Raichle, 2015).

2. The format of the equations causes readability issues, and the format is somewhat inconsistent throughout the manuscript:

2.1. I suggest avoiding the use of "cdots" to indicate scalar multiplication and to drop the temporal depdencies. Both these things appear in the first set of equations (1-6, JR model) and are slowly dropped in subsequent systems. I suggest they are removed altogether for the sake of simplicity. If necessary, cdots might be substituted by small spaces to clearly separate variables. cdots and temporal dependencies were removed.

2.2. In all equation systems, particularly eqs. 1-6 and eqs. 9-12, please use consistent justification to aid readability (i.e. all equations with the same justification so that equal symbols align consistently).

Justification for equations 1-6 and 9-12 were included.

2.3 Continuing with the first set of equations (1-6, JR model):

2.3.1 I strongly recommend expressing the system as three 2nd order differential equations rather than six 1st order equations (as it appears now), as the former is generally more readable.

Thanks for the suggestion. However, we would prefer to keep the classical formulation of the Jansen and Rit (1995) with 6 equations which is the most generally use case in literature (Grimbert & Faugeras, 2006; Spiegler et al., 2010; Forrester et al., 2019).

2.3.2 Furthermore, please use square brackets in eqs. 5,6 at the largest level, instead of parentheses as it is written now.

We used brackets at the largest level in equation 5 following Jansen and Rit (1995) and removed the parentheses from equation 6.

2.3.3 Similarly, the variable input(t) is missing the subscript i.

Included, thank you.

2.3.4 In order to improve readability, it may also be an option to substitute y0,y1,y2 for x,y,z to eliminate the subindex and avoid double subscripts. Adopting more descriptive variable names, such as pn (pynamidal neurons), ei (exc. Interneurons) or ii (inhibitory interneurons) is also an option the authors may consider.

We would prefer to keep the classical formulation by Jansen and Rit (1995).

2.3.5 Similarly, please consider changing "input" with a simpler variable name such as "I" (see also following comment).

Done, thank you.

2.4. For the second set of equations (9-12, protein dynamics), readability is also hindered, particularly due to the protracted variable names. While I understand this is done so that the variables are more descriptive, in this case it hinders the readability of the equations.

2.4.1 Hence, I strongly recommend employing simple, short variable names. For instance, use the symbol "\tau" instead of "TAU", and add a bar to the toxic variables (\bar{a\beta} and \bar{\tau}) instead of adding the termination "t". The current notation has led to typos and inconsistencies in eqs. 11 and 12, with the use of both "T" and "\tau" instead of "TAU", and in eq. 12 "TAU_t" is used instead of "TAUt".

We did not use τ for tau protein to avoid the confusion with the time constant variables in the JR NMM (τe and τi). Therefore, we will substitute the TAU expression with a capital T. Additionally, we will also substitute the toxic termination with the suggested bar. Thank you very much.

2.4.2 Please also simplify the notation for "clear", "trans" etc. (at the very least using non-italic fonts for spelled out words might be sufficient -this also applied to "input" in the previous set of equations- but I strongly recommend simplifying the notation here).

We will take the non-italic fonts suggestion while keeping the descriptive variables to help the non-mathematical reader to understand more easily the model.

2.4.3 Finally, some variable definitions are missing: the variable "syn" is not defined below that I can see, and a brief definition of q^ha and a reference to its further discussion would be helpful to avoid unnecessary confusion.

You are completely right, thank you very much. We added a description for "syn" variable in the text and a brief definition and reference to q(ha) description.

2.5. Similarly, in eqs. 14 and 15 a shorter name for "A\beta t _{damrate}" would enhance clarity.

We implemented shorter names for those variables (e.g., cq(Aβ)).

2.6. In general, the parameters introduced in each equation remain undefined. While in the first set of equations there is a reference to a table describing the parameters, this referencing is less evident in the subsequent equation sets. Hence, I suggest including brief definitions of the main parameters, or a more explicit reference to the corresponding table.

There was a reference to a table in line 196 ("see Table 2 for a description of parameters") that contains most of the parameters in equations 9 - 21.

2.7 Can \Delta ha_i (eq. 22) become negative in later stages? How is this handled in the model? \Delta ha_i cannot be negative as firing rates are always positive (both hai and hai0). Therefore, \Delta ha_i ϵ (0, inf).

3. Given the complexity of the modeling frameworks, I suggest including an initial figure with a schematic overview of the modeling framework (which may also include the SC matrix as suggested above). I suggest two panels, one for the JR model, illustrating the coupling within and between regions, and a second panel describing the different components of the BNM model and their interactions (i.e., in the same spirit for the central panel in fig. 2, but more detailed).

We have developed a figure to summarize the modeling framework. It will be implemented as the first figure of the article. Therefore, in the revised version all figures will have an updated id (+1), but we kept the original figure ids in the rest of this review to avoid confusions.

Figure 0. Modelling framework overview. A) The closed-loop neurotoxicity model integrates a model of proteinopathy dynamics and a BNM to simulate neural activity, interacting in a closed-loop: proteinopathy affects neural activity and vice-versa. B) Scheme of the JR NMM with three interacting subpopulations where triangles represent synaptic contacts between subpopulations, boxes represent the sigmoidal transformation of voltage into firing rate and coloured hexagons represent the transformation of firing rate into voltage. C) Implementation timeline for the closed-loop model. First, a baseline simulation is gathered to get a baseline firing rate. Then, the closed-loop model simulation begins by integrating the proteinopathy with dt=0.25 (years) and simulating the BNM to update hyperactivity damage with dt=1 (year). D) Pipeline to extract SC matrices. Tractography is performed on dw-MRI data, and SC matrices are extracted using a customized version of the DK atlas.

4. In line 218-219, please briefly define "He", "Cip", "Cep", as these are only defined before on a table and not the main text.

Definitions were included.

5. I am curious for the choice of temporal scales for the two models (line 260). Why is the NMM not run between each iteration of the neurotoxicity model? Have you explored the effect of the choice of timescales? Nice observation. We used these different integration timescales due to the long-lasting simulations of the BNM, that indeed they were intended to be enlarged to get further information and more accurate information regarding FC / dFC that is not explored finally in the model. We did not explore systematically the effect of the differences in integration times, but we have noticed that changing those may accelerate or slow down the rhythm of the system changes.

6. Please briefly defined "g" and "s" in line 267 since these have not been defined in the main text.

They stand for coupling factor (g), a variable that scales connection weights, and conduction speed (s), a variable that determines conduction delays. The former was described in the main text in line 171. For the latter, we have corrected a mistaken symbol used "v" instead of "s" in section 2.3, where conduction speed (s) was defined in line 160.

7. The final paragraph of section 2.5 is hard to follow. I found the expression "adjusting the model" rather unclear, and moreover the analyses described in this section were not apparent to me until after reading the results. From the results section I understood that the exploration of the effect of the extreme values for the different variables serves to characterize their effect on the functional dynamics, and to select "adjusted parameters" that best describe AD pathology. I do not understand however how are these adjusted parameters selected? Are these adjusted parameters used in figures 3 and 6? Thank you for the observation. We extended the explanation in that section for the sake of clarity: "We explored those parameter spaces to select a set of parameter values that could reproduce qualitatively the empirical observations reported in the AD continuum: the slowing of alpha frequency, and the rise and decay of relative alpha power, cellular firing rate and FC. The adjusted parameters are used here as the default values for our model and are included in Tables 1 and 2." I hope this orients the reader to answer your questions. The parameters were selected to reproduce qualitatively empirical observations reported in AD literature. And, indeed, those adjusted parameters were used in the simulations of figures 3 and 6.

8. Line 289-290, on the amyloid-beta seeding profiles, should be the first line of the subsequent paragraph. Moreover, the antero-posterior differentiation is not properly introduced in the introduction of the manuscript.

From our perspective, line 289-290 belongs to the first paragraph where we state our aims with the spatiotemporal profile assessment. The second paragraph is about the methodology we are implementing. However, we agree with the reviewer that it is not well positioned into that paragraph. We moved the sentence to the beginning of the paragraph: "We evaluated the spatial propagation of hp-tau using the Braak stages as a reference and the impact of *A*β seeding over the temporal antero-posterior differentiation in terms of firing rate and FC." Additionally, we extended the introduction to mention more explicitly the antero-posterior differentiation: "These observations suggest a temporal dissociation between anterior and posterior in the electrophysiological changes that occur in AD (Maestú et al., 2021)." 9. In section 3.1, it would be helpful to show some representative time series of the NMM variables for each of the three regimes.

We added a figure as extended Figure 1-2 showing a couple of representative time series from each of the three JR regimes.

10. The first discussion of the effect of model parameters on the phase diagrams shown in figure 1 involves He and Cep, however, this combination is not shown in the figure, which is counter-intuitive. I understand the reasoning for showing He and Cip instead, but perhaps the authors can edit the text or the figure to circumvent this issue.

Thank you very much. We tried to orient the reader not to expect a heatmap with both parameters by including a direct reference to the part of the figure where they could find the information: "the parameter candidates related to intranode excitation (i.e., He and Cep; see 1st and 2nd-3rd columns of Figure 1, respectively)." 11. In figure 1, I suggest the authors to delineate the fast and slow limit-cycle regions with some contour lines to aid readability. I am also curious for why three different colormaps are used in this figure.

We believe that contour lines may obstacle the reading and understanding of heatmaps as the transitions from one limit cycle to another are abrupt at certain points but smoother and more progressive in others. Additionally, three colorscales were used to represent different aspects of the model's output: one for frequency peak, another for spectral power and one more for cellular firing rate.

12. In lines 369-371 it is mentioned that "the firing rate kept rising monotonically as inhibition lowered, while the frequency peak and power tended to be reduced" however I find it difficult to conclude this from the colormaps alone. A supplementary figure showing the 1D curves resulting from varying one parameter only (Cip in this case), corresponding for instance to the red point marked in the figure, would suffice to address this issue.

We included an additional figure showing 2D curves for each measure and parameter as extended Figure 1-1.

13. In lines 391-392 it says that "the BNM integrates the effects of those proteins over neural tissue, which in turn influences the production of Abeta based on firing rate." Doesn't neural activity affect tau spreading as well? Thank you very much, you are completely right. We completed that sentence.

14. In figure 2, the authors show the evolution of different variables of the neurotoxicity model over time, averaged over brain regions. However different regions have different temporal profiles, hence I think it would be useful to show here some individual curves for different regions, in order to illustrate the spatial heterogeneity. Or does this figure correspond to the dynamics simulated only for one region? Could the authors clarify this point? In any case, a figure illustrating the spatial heterogeneity would be a valuable addition to the manuscript in my opinion.

Yes, the dynamics shown in figure 2 correspond to the averaged dynamics for the whole simulated brain. We developed and included a new figure 2 showing spatial heterogeneity grouped by areas including frontal, parietal, temporal and the cingulum.

15. Also in figure 2, I am really not a fan of the style used to show the different scales in panel C. I would suggest plotting normalized values (from 0 to 1) and indicating the minimum and maximum values for each variable in the caption. Otherwise, changing the grid on the y-axis so that the ytick-labels correspond to more extreme values, and perhaps adding also a vertical arrow for each of the variable scales, can help readability.

Figure 2 has been updated.

16. In the result section 3.2 (and in general throughout the results) it would help to refer back to the relevant equations from the methods section. Also please use the same notation as in the methods, e.g. in figure 2B and 2E. I also think there is a typo in figure 2C such that "Cie" and "Cee" should respectively be "Cip" and "Cep".

Thank you very much. We included a reference to the methods' equations in the section 3.2.

17. The effect of the limits of the parameters (figure 4) is discussed clearly in section 3.4. However, it is also mentioned in sections 3.2 (paragraph starting on line 408) and 3.3 (starting line 423). Rather than helpful, I found this repetition confusing.

You are right, this early reference to figure 4 might be more misleading than explicative. We will remove those early references.

17.1 Furthermore, figure 4 is currently mentioned before figure 3, and it is not clear whether the results of figure 4 affect figure 3 (i.e. to what parameter configuration does figure 3 correspond to)? The parameter values used in figure 3 correspond to the highlighted red values of the parameter spaces in figure 4. We rephrased figure 4 caption to make clearer this connection.

17.2 How were the values of the parameters "adjusted"? This question connects to comment number 7. We have extended the methods explanation for the parameter adjustment.

17.3 I think the manuscript would benefit from a more clear separation between the different sections, by i.e. removing the aforementioned paragraphs, and including in section 3.4 or the corresponding methods section some more detail on how the parameters were adjusted. It is not clear to me whether that would require inverting the order of some result sections and/of figures 3 and 4, but in any case a separate section discussing the parameter "adjusting" analysis and the phase diagrams in figure 4 would be helpful.

The reference of figure 4 in section 3.3 was mistaken, it was substituted with figure 3. By removing early references to figure 4, we believe that the text is useful for contextualizing the findings of each section.

17.4 Similarly, an intuitive explanation of the effect of the limits of the model parameters should be included already in the corresponding methods section.

We hope the new explanation of the model fitting in the methods section makes clearer the effect of the limits of change in the model.

18. I recommend that the authors consider the inclusion of an additional schematic figure at the end of section 3.4. This figure could serve as a concise visual summary of the primary findings from the analyses, particularly highlighting which parameters can lead to each of the distinct Alzheimer's disease pathologies under consideration, according to the model.

We believe that an additional figure at that point could become confusing.

19. Why is the study done in figure 7 performed for amyloid beta and not tau? Thanks for the comment. We have recalculated this study including hp-tau seeding strategies and they will be included in the manuscript. Indeed, results showed interesting relationships. The result section and captions have been updated.

20. I found a few spelling errors and typos:

20.1. Line 55: the reference "Maestú et al." is missing parentheses.

We changed that reference following a previous comment and added parentheses. Thank you! 20.2. "TVB" in line 151, which I assume stands for the virtual brain, is not defined We rewrote it as The Virtual Brain.

20.3. In line 170, please write "standard deviation" instead of "std".

That line was rephrased for clarity.

20.4. There seems to be an extra "\newline" inserted before some of the equations (eq. 1, eq. 9, eq. 14). Please remove this.

Completely right, thank you very much.

20.5. The phase variable "phi" in eq. 23 is not defined.

Definition included.

20.6. In line 269, I believe it should say "functional connectivity" instead of "PLV"? Sure, changed, thank you.

21. The literature review is extensive, but I recommend considering also the following studies:

Regarding tau spreading:

21.1. Schoonhoven, Deborah N., et al. "Tau protein spreads through functionally connected neurons in Alzheimer's disease: a combined MEG/PET study." Brain (2023): awad189.

Cited in discussion.

21.2. Lamontagne-Kam, Daniel, et al. "Implication of tau propagation on neurodegeneration in Alzheimer's disease." Frontiers in Neuroscience 17 (2023).

Thank you very much. We included a paragraph in discussion associated with this article: "In this work, we used the entorhinal cortex as the seeding region for hp-tau propagation. However, recent studies suggest that the classical Braak staging of tau pathology in AD \citep{Braak1991} may not offer a comprehensive view and may disregard alternative tau epicentres and propagation trajectories \citep{Franzmeier2020, LamontagneKam2023}. From the epicentres, hp-tau spreads to functionally connected regions \citep{Schoonhoven2023} producing different network disruptions and clinical profiles. In a study by \cite{Vogel2021}, they identified four distinct tau propagation patterns, each capable of explaining between 18 to 33\% of the cases. We believe that our framework poses a good opportunity to advance the understanding and prediction of personalized tau propagation trajectories. Our approach considers not only structural and functional connectivity networks but also accounts for the progressive changes in neural activity due to proteinopathy." Regarding the link between network hyperexcitability / hyperactivity and network hypersynchrony:

21.3. Stam, C. J., et al. "Network Hyperexcitability in Early Alzheimer's Disease: Is Functional Connectivity a Potential Biomarker?." Brain Topography (2023): 1-18.

Cited in discussion.
