# Supplementary figures and images for: A Multiscale Closed-Loop Neurotoxicity Model of Alzheimer’s Disease Progression Explains Functional Connectivity Alterations
Source: eNeuro. 2024 Apr 12;11(4):ENEURO.0345-23.2023. doi: 10.1523/ENEURO.0345-23.2023 (PMC11026343; doi:10.1523/ENEURO.0345-23.2023)

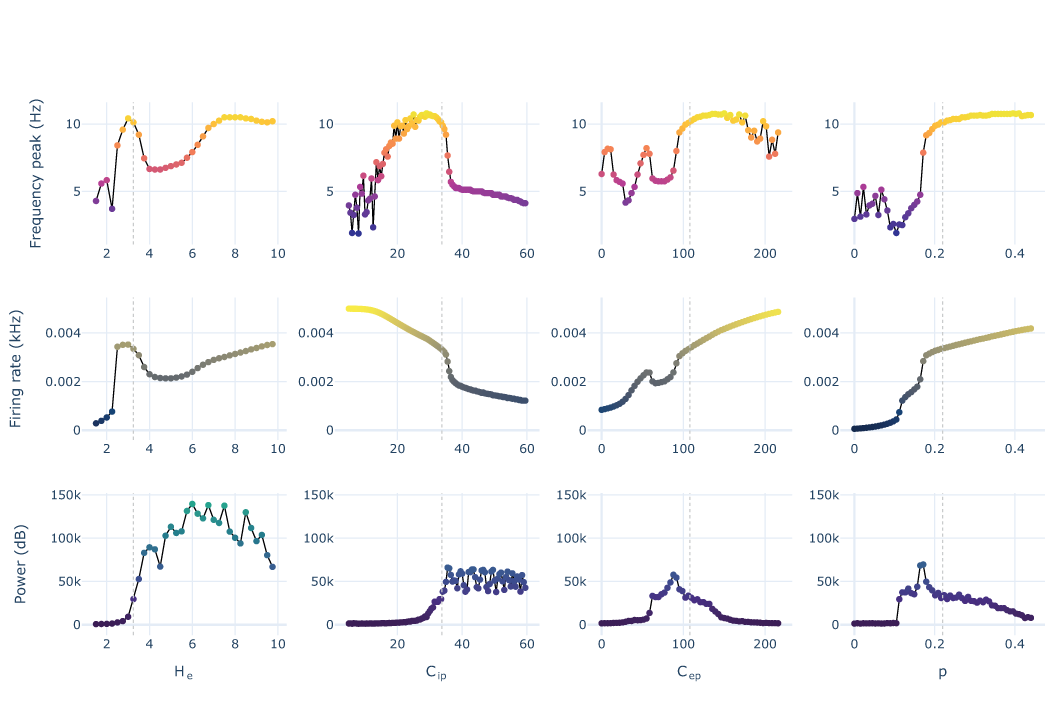

Supplement: Figure 2-1 — 2D representations of single node experiments varying only one parameter at a time (i.e., He, Cip, Cep, p). Dashed lines showing the default parameter value. Markers' colour scales are the same as in the heatmaps of Figure 2. Download Figure 2-1, TIF file. [file eneuro-11-ENEURO.0345-23.2023-s002.tif]

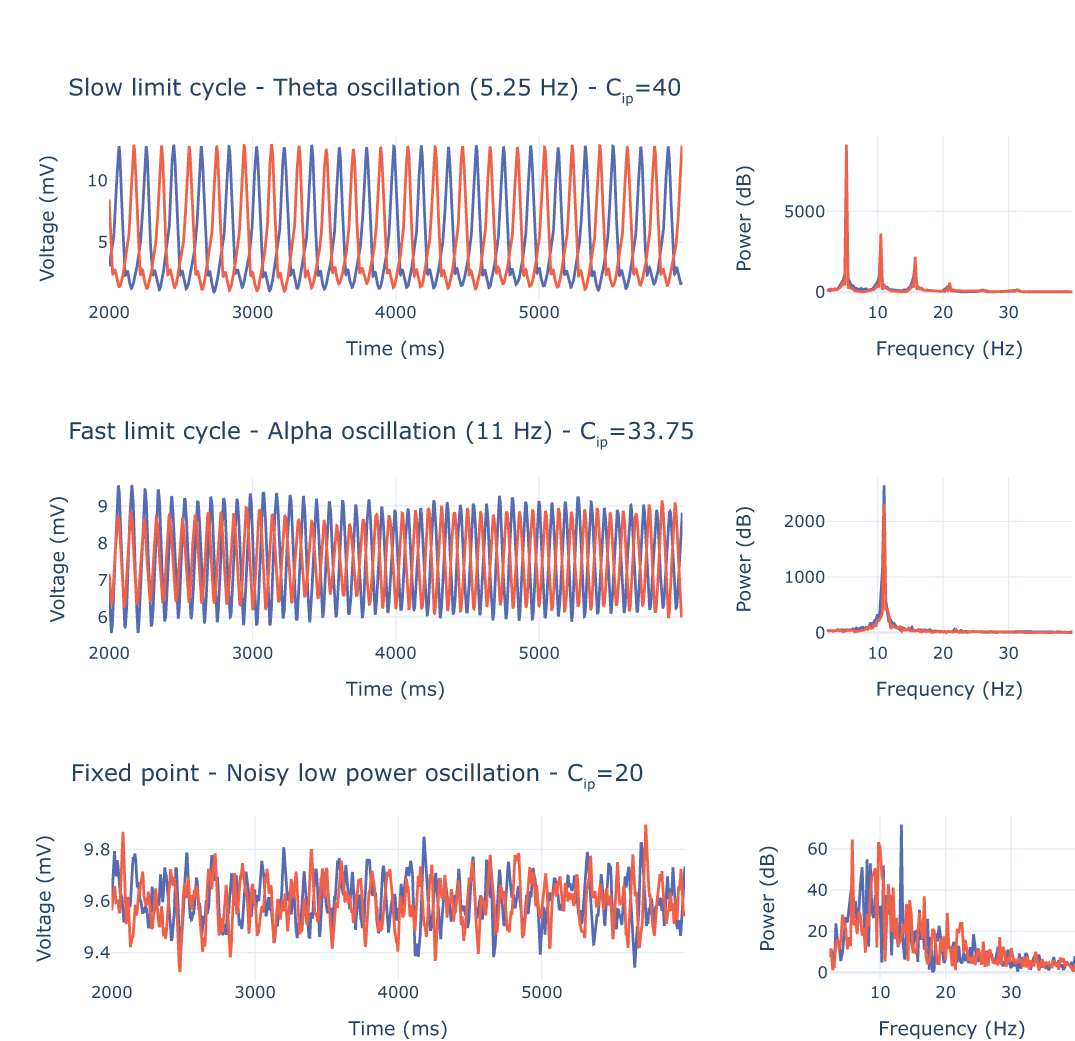

Supplement: Figure 2-2 — Timeseries and spectra for three different regimes shown in Figure 2: slow limit cycle, fast limit cycle and fixed point state. Two traces from independent simulations are shown. Download Figure 2-2, TIF file. [file eneuro-11-ENEURO.0345-23.2023-s003.tif]

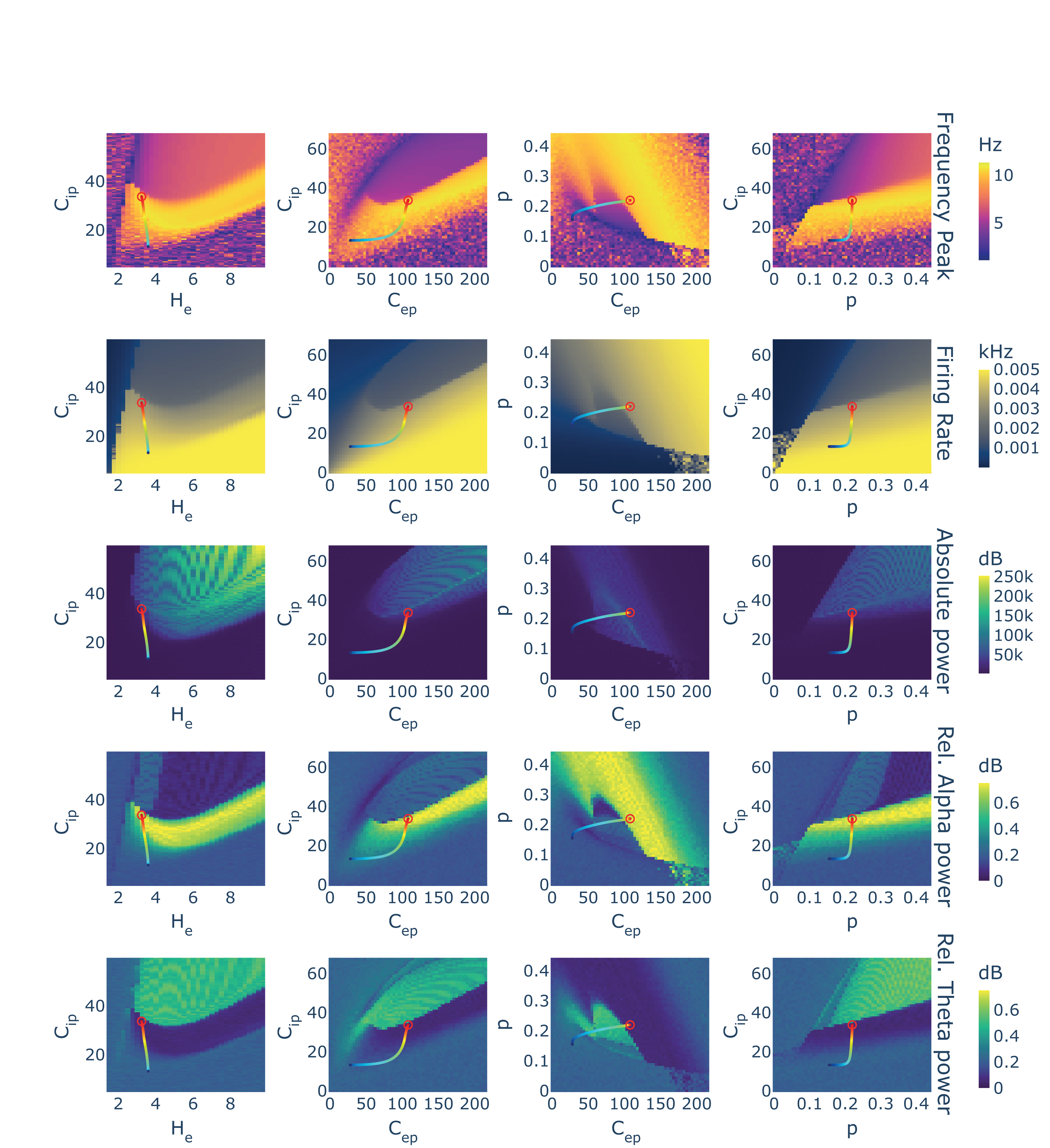

Supplement: Figure 3-1 — Parameter trajectories of the simulated closed-loop model with default parameters on heatmaps from single node experiments. Trajectories are represented as curves with varying colours. The colour represents time: starting in red and ending with blue. Note that heatmaps are extracted from single-node simulations in which just two parameters are varied at a time, while the trajectories imply a 4-dimensional parameter change. Therefore, the underlying heatmaps should be interpreted just as an orientation of what might happen when changing parameters in one direction. Download Figure 3-1, TIF file. [file eneuro-11-ENEURO.0345-23.2023-s004.tif]

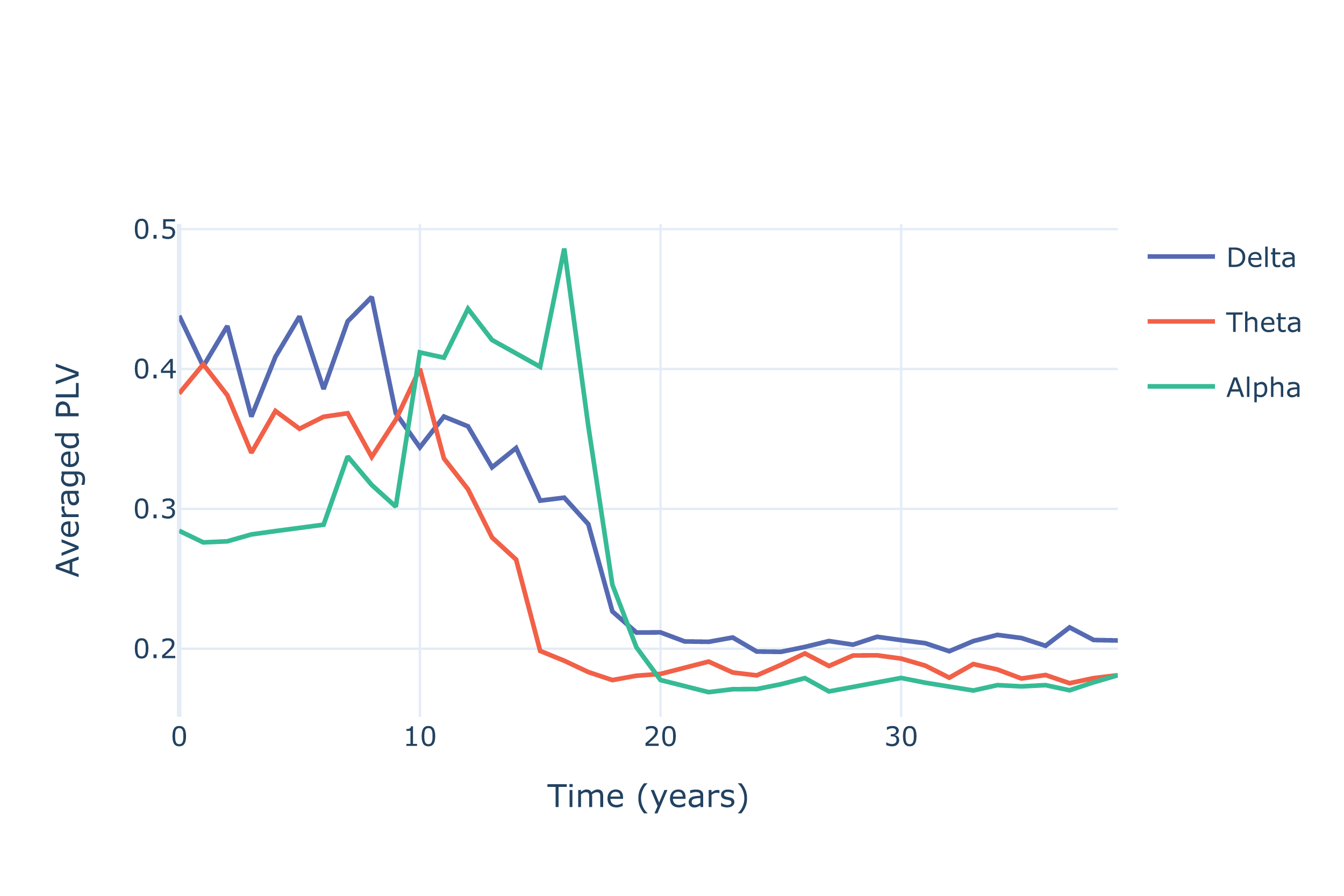

Supplement: Figure 4-1 — Averaged FC (PLV) of the simulated closed-loop model with default parameters for different frequency bands: delta (2 - 4 Hz), theta (4 - 8 Hz) and alpha (8 - 12 Hz). Download Figure 4-1, TIF file. [file eneuro-11-ENEURO.0345-23.2023-s005.tif]

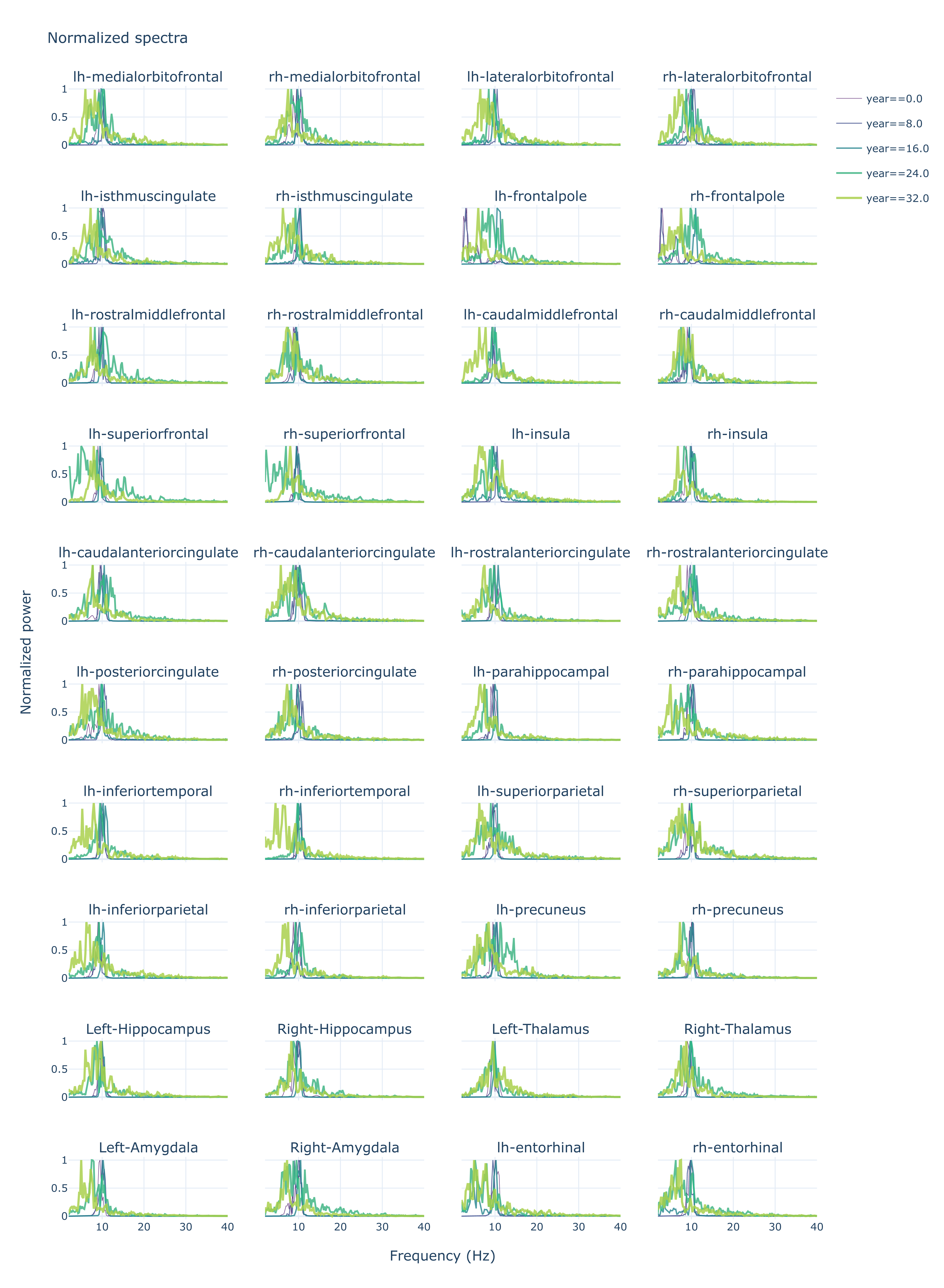

Supplement: Figure 4-2 — Normalized spectra per region over time (yrs.). Note the transition of all spectra to more noisy states with lower frequency peaks. Download Figure 4-2, TIF file. [file eneuro-11-ENEURO.0345-23.2023-s006.tif]

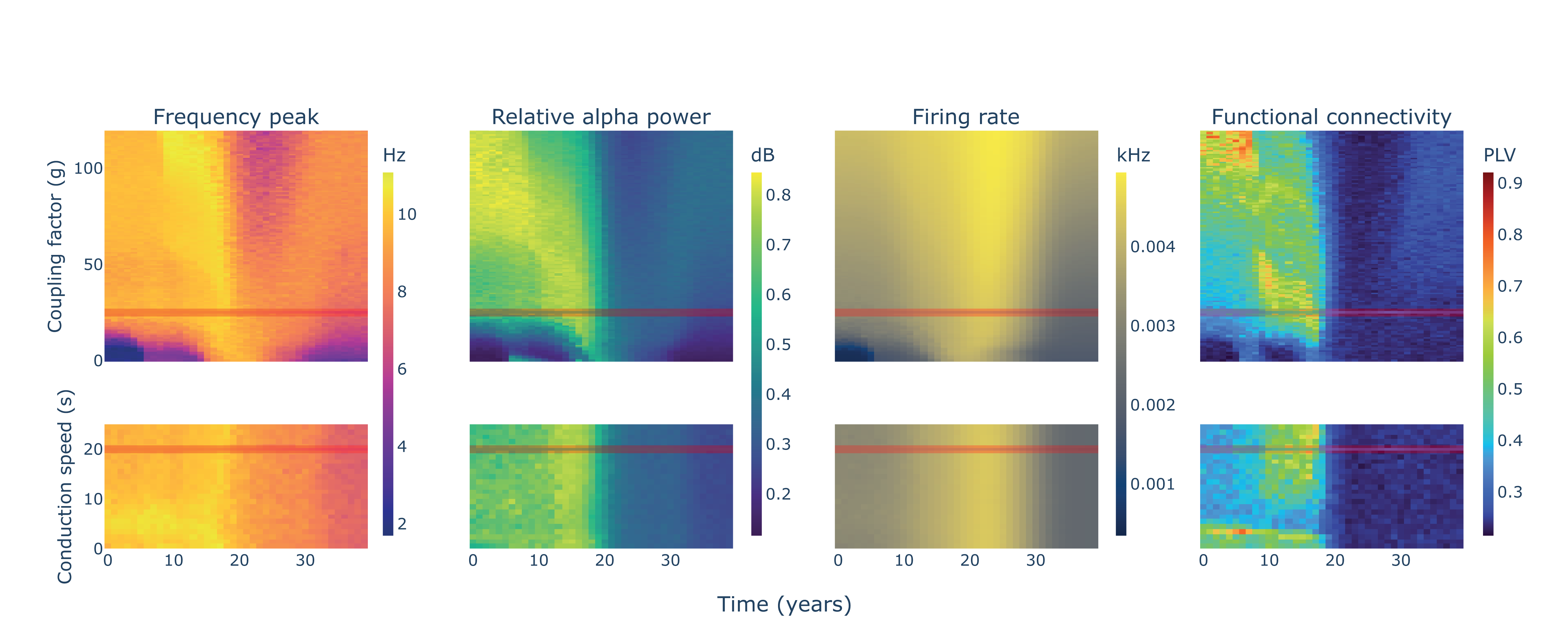

Supplement: Figure 5-1 — Parameter spaces to select working point. Selected parameters were g=25 and s=20 m/s (see red highlights). Note how the rising of g leads to a situation in which no FC rise is observed, similar to the reduction of s, due to high early FC levels. Also, lowering g leads to the prebifurcation regime of the JR NMMs, a situation in which the spectral frequency peak lowers towards the delta band. Download Figure 5-1, TIF file. [file eneuro-11-ENEURO.0345-23.2023-s007.tif]

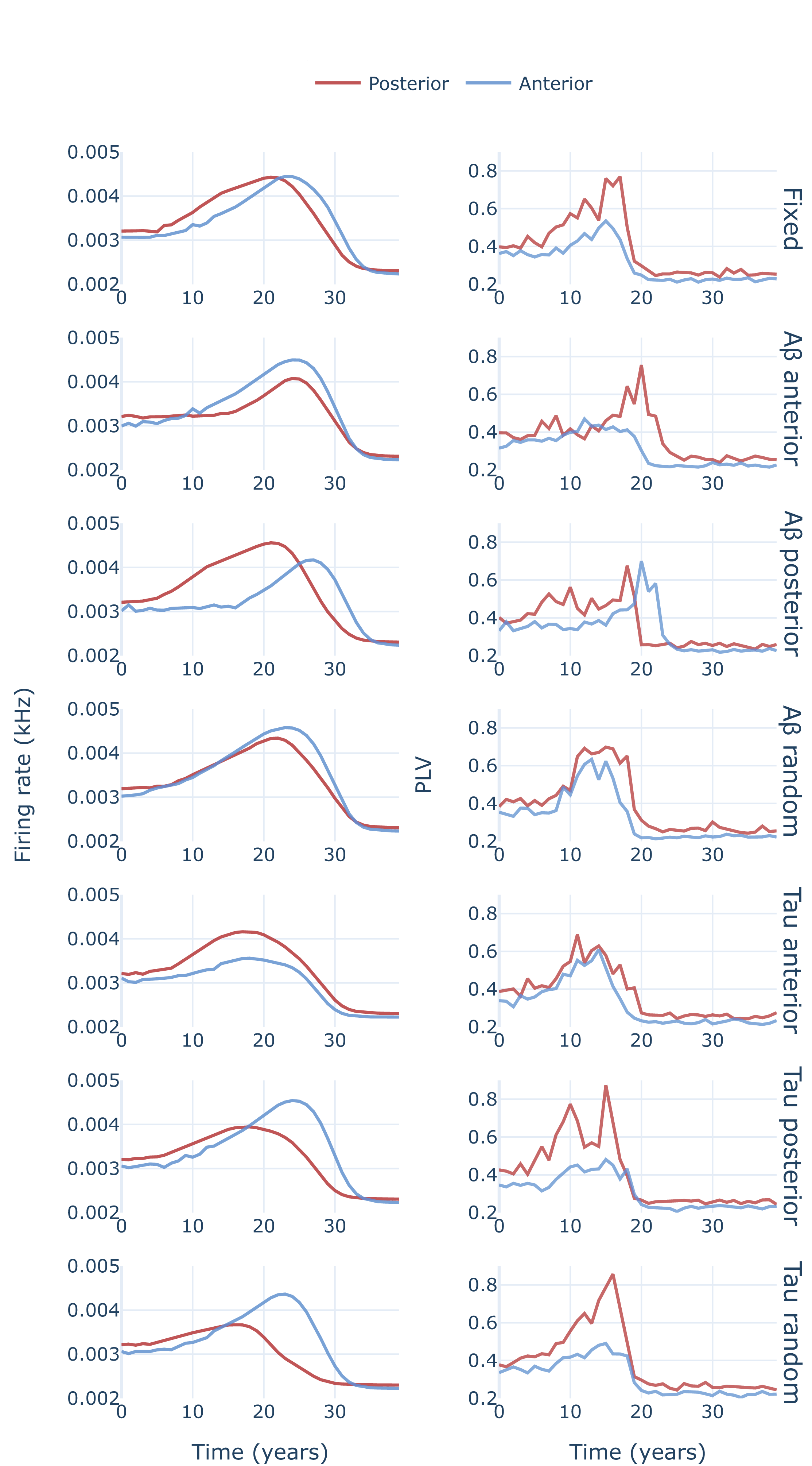

Supplement: Figure 8-1 — Samples of the evolution for firing rate (left column) and FC (i.e., PLV; right column) in the antero-posterior differentiation experiments. In rows, each of the four seeding implemented strategies. The effect on FC is limited to a temporal shift of the curves, however, the seeding affects the level of hyperactivity reached by the anterior or posterior regions. Download Figure 8-1, TIF file. [file eneuro-11-ENEURO.0345-23.2023-s008.tif]
